# Development of a Predictive Statistical Pharmacological Model for Local Anesthetic Agent Effects with Bayesian Hierarchical Model Parameter Estimation

**DOI:** 10.3390/medicines10110061

**Published:** 2023-11-15

**Authors:** Toshiaki Ara, Hiroyuki Kitamura

**Affiliations:** 1 Department of Pharmacology, Matsumoto Dental University, 1780 Gobara Hirooka, Shiojiri 399-0781, Nagano, Japan; 2 Matsumoto Dental University Hospital, 1780 Gobara Hirooka, Shiojiri 399-0781, Nagano, Japan

**Keywords:** local anesthetic agent, statistical model, Bayesian hierarchical model, computer simulation, alternatives to animal experiments

## Abstract

As an alternative to animal use, computer simulations are useful for predicting pharmacokinetics and cardiovascular activities. For this purpose, we constructed a statistical model to simulate the effects of local anesthetic agents. To train the model, animal experiments were performed on 6-week-old male Hartley guinea pigs. Firstly, the guinea pigs’ backs were shaved, then local anesthetic agents were subcutaneously injected, with subsequent stimulation of the anesthetized site with a needle six times at regular intervals. The number of reactions (score value) was counted. In this statistical model, the probability of reacting to needle stimulation was calculated using the elapsed time, type of local anesthetic agent, and presence or absence of adrenaline. Score values were assumed to follow a binomial distribution at the calculated probability. Parameters were estimated using the Bayesian hierarchical model and Hamiltonian Monte Carlo method. The predicted curves using the estimated parameters fitted well the observed animal values. When score values were predicted using randomly generated parameters, the median of duration was similar between animal experiments and simulations (Procaine: 55 min vs. 50 min, Lidocaine: both 60 min, and Mepivacaine: both 85 min). This approach effectively modeled the effects of local anesthetic agents. It is possible to create the simulator using the parameter values estimated in this study.

## 1. Introduction

As animal welfare becomes increasingly important, reducing the number of experimental animals is desirable. In identifying alternatives to animal experiments, the 3Rs are an effective strategy. They are replacement (directly replace or avoid the use of animals), reduction (obtain comparable information levels from fewer animals), and refinement (minimize or eliminate animals’ pain and distress, improving their welfare) [1]. As an alternative to animal experiments, computer simulations are used in areas including pharmacokinetics [2,3], organ bath systems, and cardiovascular systems (Strathclyde Pharmacology Simulations package: OBSim, RatCVS and Virtual Cat) [4]. Free downloadable computer software packages for teaching pharmacology are summarized in a recent review [5]. In simulators for pharmacokinetics, drug blood concentration is calculated by solving ordinary differential equations that follow the compartment model numerically.

Animal experiments are a long-used educational tool to evaluate the effect of local anesthetic agents in the practice of pharmacology. One of the animal experiments is to inject multiple local anesthetic agents into the back of guinea pigs and examine the number of times they respond to needle stimulation. Animal experiments using guinea pigs have the advantage that the effects of multiple drugs can be investigated simultaneously. We have used this method to investigate the effects of several local anesthetic agents [procaine (Pro), lidocaine (Lid), mepivacaine (Mep), bupivacaine (Bup), and lidocaine with adrenaline (Lid + Adr)] in terms of their strength and duration in the practice of pharmacology. Generally, local anesthetic agent duration is determined by peripheral vasodilation and lipid solubility [6,7]. As many local anesthetic agents dilate peripheral blood vessels, these drugs migrate into blood vessels and disappear from the administration site. Therefore, the duration of action of the drug is shortened. Moreover, as drugs with high lipid solubility can easily pass through cell membranes, these drugs have tendencies toward long duration. Pro and Lid both induce peripheral vasodilation, while Mep induces less vasodilation [6]. In contrast, Bup induces vasoconstriction [6,7]. In addition, the lipid solubility of Bup is high. These characteristics rationalize the short durations of Pro and Lid, the relatively long duration of Mep, and the very long duration of Bup. Vasoconstrictors such as adrenaline or felypressin are very often added to local anesthetics. The purpose of adding vasoconstrictors is to prolong the duration of action of local anesthetic agents. By constricting the blood vessels, the local anesthetic slows its entry into the blood vessels, thus remaining in the tissues and prolonging its duration of action. The purpose of the practical training is to confirm the above contents through animal experiments.

From an animal welfare viewpoint, it is desirable to replace animal experiments for local anesthetic agents with computer simulations. There are many commercially available simulators for technical training of local anesthesia. However, to our knowledge, there is no simulator aimed at pharmacological effects such as intensity or drug effect duration. We believe that creating a simulator for these purposes will greatly contribute to reducing the number of experimental animals. Therefore, in this study, we developed a computer simulation model for training on local anesthetic agents. To achieve this, we constructed new statistical models and estimated drug parameters.

For modeling and estimation of parameters, we used the Bayes hierarchical model and the Hamiltonian Monte Carlo (HMC) method. Research using Bayesian hierarchical models often analyzes the influence of factors by creating statistical models. For example, Bayesian hierarchical models are used in many areas such as clinical trials [8,9,10,11], animal experiments [12,13,14], and genetics [15,16]. Regarding the creation of statistical models for simulators, there are models for reproducing the movement of the myocardium [14,17]. However, there are no models for local anesthetic agents.

When score values were predicted using the estimated parameters by computer simulation, these values were similar to those from animal experiments. Therefore, the statistical model in this study provides a novel theoretical background to create a simulator for local anesthetic agents used in the practice of pharmacology. It therefore becomes possible to create the simulator using parameter values estimated in this study.

## 2. Materials and Methods

### 2.1. Animals and Drugs

Six-week-old male Hartley guinea pigs were purchased from Japan SLC (Shizuoka, Japan). The guinea pigs were housed up to 3 per cage (350 × 420 × 200 mm). All guinea pigs were housed in a specific-pathogen-free facility at Matsumoto Dental University at 24 ± 2 °C and 50–60% humidity with a 12 h light/dark cycle, and had free access to sterilized water and a normal diet (Labo G Standard, SLC). Following 1 week adaptation, guinea pigs were used for the experiments. All guinea pigs were euthanized using 1% pentobarbital sodium for intraperitoneal anesthesia. According to the guidelines of the Animal Management Committee of Matsumoto Dental University, all procedures for animal care were approved and carried out.

The 1% Procaine hydrochloride (Pro) was purchased from Fuso Pharmaceutical industries (Osaka, Japan). The 1% Lidocaine hydrochloride (Lid) and 1% Lidocaine hydrochloride with 1/100,000 adrenaline (Lid+Adr) were purchased from AstraZeneca (Osaka, Japan). The 1% Mepivacaine hydrochloride (Mep) was purchased from Mylan Seiyaku (Tokyo, Japan). The 1% Bupivacaine hydrochloride (Bup) was purchased from Aspen Japan (Tokyo, Japan).

### 2.2. Data in Animal Experiments

Fourteen to sixteen guinea pigs per year were used in the practice. Saline (as a control) and 5 drugs were injected to each guinea pig. All results from 2019, 2021, and 2022 were used. The 2020 results were not used as results of Pro testing yielded unacceptably high variability. The total number of animals was 51.

**Figure 1:**
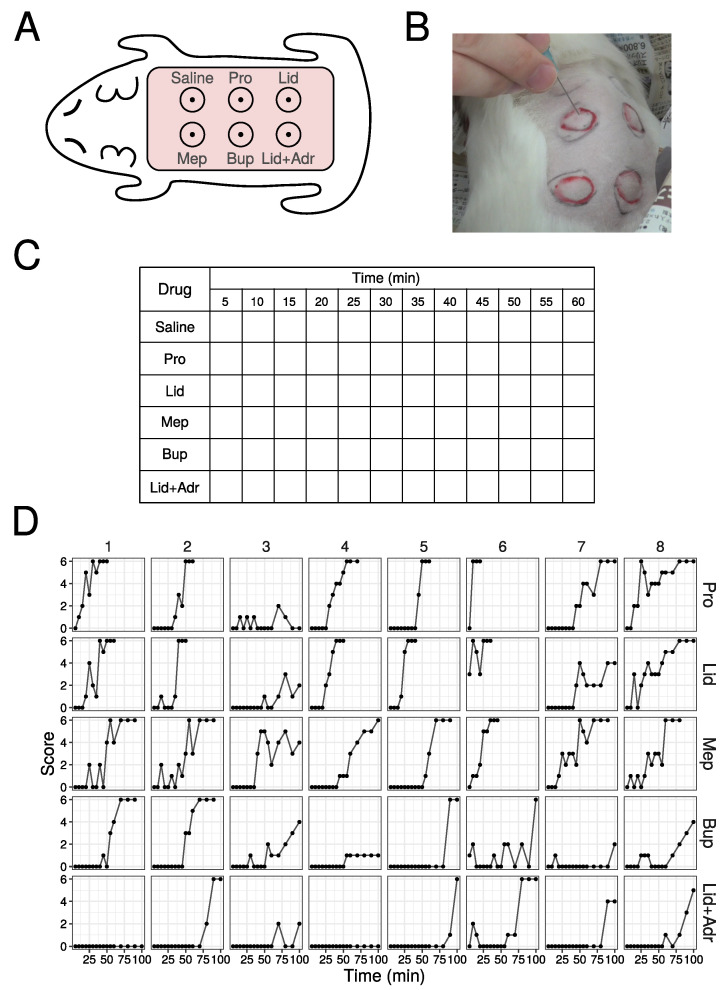
Raw data from animal experiments in the practice of pharmacology. (**A**) Schema of injection site of local anesthetic agents. (**B**) Photography of stimulation by needle. (**C**) Table used in practice. (**D**) Score values in the animal experiments. First eight data out of 51 are shown (All data are shown in Figure S1).

The method of training is performed as follows: (1) shave the hair on the back of the guinea pig; (2) inject 0.1 mL of saline and 5 drugs intradermally (Figure 1A); (3) each injection site papule is enclosed in a circle marked by a magic marker; (4) stimulate 6 times at each papule with needle (Figure 1B), with the number of the skin contractions counted, defining this number as the score (minimum is 0, and maximum is 6), and scores are then recorded in the example Table (Figure 1C); (5) stimulate at 5 min interval up to 120 min. When a score of 6 is obtained three times in a row, finish the stimulation and define that time as the duration.

This experiment was approved by the Animal Management Committee of Matsumoto Dental University (No. 356 in 2019, No. 396 in 2021, and No. 413 in 2022).

### 2.3. Statistical Model by Bayesian Hierarchical Model

**Figure 2:**
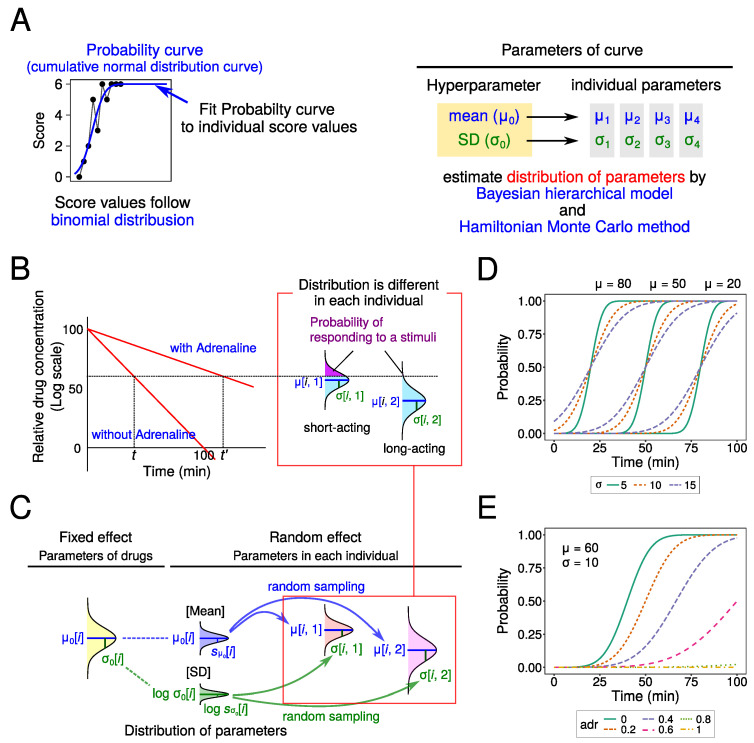
Statistical model in this study. (**A**) Schema of modeling process. Fit probability curve (cumulative normal distribution curve) to score values, which follow binomial distribution. The distribution of Hyperparameter (mean and SD of each drug) and parameters in each individual were estimated by Bayesian hierarchical model and Hamiltonian Monte Carlo method. (**B**) Calculation of reaction probability to needle stimulation. Upper probability of normal distribution in each drug is used (violet region). (**C**) The fixed effect model and random effect model used in this study. In the fixed model, the distribution of each drug is assumed to be a normal distribution (mean: $\mu_{0}\left[i\right]$, SD: $\sigma_{0}\left[i\right])$). In the random effect model, μ[i,j] is randomly sampled from normal distribution, and logσ[i,j] from lognormal distribution. (**D**,**E**) The effects of parameters on probability curve: change in μ and σ (**D**), and adr (**E**).

In this study, we tried to fit cumulative normal distribution curves to the raw data as the probability of responding to a stimulus (i.e., probit model). And we assumed that score values were determined by a binomial distribution in their probabilities (Figure 2A). As the center and slope of the curve are different among drugs, we estimated the parameters [mean ($\mu_{0}$) and SD ($\sigma_{0}$)] for each drug. Also, we estimated the parameter values indicating the effect of adrenaline (adr). As the main purpose is to perform computer simulations, it is necessary to determine the distribution of these parameters in order to set the parameter values with random numbers in the simulation. Therefore, we estimated the SD of these parameters ($s_{\mu_{0}}$ and $s_{\sigma_{0}}$).

We assumed two statistical models with or without offset value (*d*), which means individual difference. The assumptions used are described below (Figure 2B,C). Drug parameters were estimated using a Bayesian hierarchical model and the Hamiltonian Monte Carlo method [18].

(1) Drug concentration in local tissues decreased exponentially. This concentration was determined by elapsed time (*t*) and the presence or absence of adrenaline ($\mathrm{adr}\times V_{\mathrm{adr}}$) (Equation (1)). As adrenaline constricts blood vessels, the local anesthetics agent does not enter blood vessels and stays in local tissues. When adrenaline was present, therefore, the rate of decrease in local concentration became smaller (the slope was decreased). As this is a mathematically indefinite problem, the values of two parameters were fixed. Initial log concentration and slope were set to 100 and −1, respectively.
(2) The probability of reacting to needle stimulation was determined by drug concentration and type of local anesthetic agent (normal distribution defined by μ[i,j] and σ[i,j]) (Equation (2)). The upper probability of normal distribution was calculated (violet area in Figure 2B). The number of reactions to stimulation (score value, Score[i,j]) followed a binomial distribution at this probability (Equation (3)).
(3) The parameters (μ[i,j] and σ[i,j]) for distributions of each drug and individual followed normal and lognormal distributions, respectively (Figure 2C). In Model 1, μ[i,j] followed a normal distribution in which mean and SD are $\mu_{0}$ and $s_{\mu_{0}}$, respectively (Equation (4)). In Model 2, as the overall local anesthetic agent effect varied among individuals, the parameter d[j] as the offset value was added to the mean of the distribution (used Equation (5) instead of Equation (4)). As σ[i,j] must be positive, σ[i,j] was assumed to follow a lognormal distribution, in which mean and SD are $\log\sigma_{0}\left[i\right]$ and $\log s_{\sigma_{0}}\left[i\right]$, respectively (Equation (6)), in both models.
(4) Lastly, the following distributions were assumed for the prior distribution of parameters. $\mu_{0}\left[i\right]$ followed the Cauchy distribution (Equation (7)). $s_{\mu_{0}}\left[i\right]$ followed the half-Cauchy distribution (Equation (8)). $\log\sigma_{0}\left[i\right]$ and d[j] followed a normal distribution (Equations (9) and (10)). $\log s_{\sigma_{0}}\left[i\right]$ and adr followed uniform distributions (Equations (11) and (12)).

Assumed statistical model:

(1) $$\begin{array}{c} \mathrm{Concentration}=100-\left(1-\mathrm{adr}\times V_{\mathrm{adr}}\right)t \end{array}$$

(2) $$\begin{array}{c} p\left[i,j\right]=1-\Phi \left(\frac{\mathrm{Concentration}[t]-\mu [i,j]}{\sigma [i,j]}\right) \end{array}$$

(3) $$\begin{array}{c} \mathrm{Score}[i,j]∼Bi(p[i,j],6) \end{array}$$

(4) $$\begin{array}{c} \mu \left[i,j\right]∼\mathrm{Normal}\left(\mu_{0}\left[i\right],s_{\mu 0}\left[i\right]\right)\;(\mathrm{in}\;\mathrm{Model}\;1\;\mathrm{only}) \end{array}$$

(5) $$\begin{array}{c} \mu \left[i,j\right]∼\mathrm{Normal}\left(\mu_{0}\left[i\right]+d\left[j\right],s_{\mu 0}\left[i\right]\right)\;(\mathrm{in}\;\mathrm{Model}\;2\;\mathrm{only}) \end{array}$$

(6) $$\begin{array}{c} \sigma \left[i,j\right]∼\mathrm{LogNormal}\left(\log\sigma_{0}\left[i\right],\log s_{\sigma_{0}}\left[i\right]\right) \end{array}$$

Prior distributions of parameters:

(7) $$\begin{array}{c} \mu_{0}\left[i\right]∼\mathrm{Cauchy}\left(50,20\right) \end{array}$$

(8) $$\begin{array}{c} s_{\mu_{0}}\left[i\right]∼\mathrm{HalfCauchy}\left(0,1\right) \end{array}$$

(9) $$\begin{array}{c} d[j]∼\mathrm{Normal}(0,20)\;(\mathrm{in}\;\mathrm{Model}\;2\;\mathrm{only}) \end{array}$$

(10) $$\begin{array}{c} \log\sigma_{0}\left[i\right]∼\mathrm{Normal}\left(2.5,1\right) \end{array}$$

(11) $$\begin{array}{c} \mathrm{adr}∼\mathrm{Uniform}(0,1) \end{array}$$

(12) $$\begin{array}{c} \log s_{\sigma_{0}}\left[i\right]∼\mathrm{Uniform}\left(>0\right) \end{array}$$

where

*i* = 1, 2, 3, 4 (Number of drugs)
*j* = 1, 2, …, 51 (Number of individuals)
*V*${}_{adr}$ is the dummy variable (0 when adrenaline is absent, 1 when adrenaline is present).
*t* is time after administration (minute).
Φ is the cumulative distribution function for the normal distribution.
*Bi* is the probability mass function for the binomial distribution.

In the fixed effect model, two parameters ($\mu_{0[i]}$ and $\sigma_{0}\left[i\right]$) were assumed as drug-intrinsic values: $\mu_{0}\left[i\right]$ and $\sigma_{0}\left[i\right]$ are the mean and SD in a normal distribution, respectively. For the random effect model, μ[i,j], σ[i,j], $\log s_{\mu_{0}}\left[i\right]$, and $\log s_{\sigma_{0}}\left[i\right]$ are assumed to reflect differences among individuals: μ[i,j] and σ[i,j] are the mean and SD of distribution in each drug and individual.

### 2.4. Estimation of Parameters by the Hamiltonian Monte Carlo Method

The Hamiltonian Monte Carlo (HMC) method was performed in Stan [19] to obtain the posterior distributions for the parameters of interest. Parameter estimation was performed using R [20] and the rstan package [21]. In this study, the parameter values were estimated given the following: a chain number of 4, iteration number (including burn-in) of 10,000 for each chain, burn-in set to 2000, and a saving sample period of 10. Fitted models were compared by the leave-one-out cross-validation information criterion (loo-ic) [22] and widely applicable information criterion (WAIC) [23] methods using the loo package [24] for R.

### 2.5. Computer Simulation

Simulations were performed as follows. Parameters (d[j], μ[i,j], and σ[i,j]) were generated by a random number generator following a normal or lognormal distribution, respectively. Probability was calculated by elapsed time and parameter values. Then, score values were determined by a random number generator following a binomial distribution for this probability. This operation was repeated 100 times.

### 2.6. Comparison of Local Anesthetic Agent Duration between Raw and Simulation Data

To compare the median of duration times for local anesthetic agents between raw and simulated data, survival analysis using the survival package [25] for R was used. In the case that local anesthetic agent effect did not subside within the measurement time (maximum 100 min for raw data and 120 min for simulations), the data were dealt with as censored data.

### 2.7. Programing Codes Used in This Study

The codes (R and Stan) used in this study are included in the supplemental_data folder of the Supplemental Data.

## 3. Result

### 3.1. Raw Data Using Animals

Raw data from 8 out of 51 animals are shown in Figure 1D (All data are shown in Figure S1). As time passed, the score values increased for many individuals. The local anesthetic agent effect subsided in the order of Pro, Lid, Mep, Bup, and Lid + Adr. The combined Lid + Adr effect duration was substantially longer than that of Lid alone, with the effect of Lid + Adr, for almost all individuals, active until the end of measurement. For several drugs and individuals, scores fluctuated up and down, while in several individuals the measured effect remained low. No adverse event was observed in this study.

### 3.2. Effect of Parameters on Probability Curve in This Model

First, the effect of two parameters (μ and σ) on the probability curve shape was examined. When the value of μ is large, the probability curve was shifted to the left (Figure 2D). This indicates a short period of effect for the local anesthetic agent. Moreover, in this model, the time for a probability of 0.5 was calculated as 100−μ.

For small values of σ, the resulting slope of the curve was large. This indicates a rapid and complete dissipation of the local anesthetic agent effect after it begins to diminish. In contrast, a large value of σ results in a small slope for the curve, indicating a gradual dissipation of the local anesthetic agent effect.

Next, the effect of adrenaline (adr) was examined. With large values of adr, the curve was shifted to the right, and the slope of curve becomes large (Figure 2E).

### 3.3. Estimation of Parameters

Mean values of posterior distribution of parameters estimated by the HMC method are listed in Table 1, Table 2 and Table 3. Parameters for all individuals are listed in the Supplemental Data. We confirmed parameter convergence by trace plot (Figure S2). Moreover, the $\hat{R}$ was less than 1.01, and the effective sample size was at least 1000 for both models.

As listed in Table 1, the estimated parameter values involved in the fixed effect model ($\mu_{0}$, $\sigma_{0}$, and adr) were similar between both Model 1 and Model 2. Moreover, the value of Pro was largest in $\mu_{0}$, and decreased in order of Lid, Mep, and Bup for both Model 1 and Model 2. This order of $\mu_{0}$ is opposite to that for the clinically observed local anesthetic agent duration.

As listed in Table 2 and Table 3, the estimated values of μ[i,j] and σ[i,j], fitted to each drug and individual and involved in the random effect model, were similar for both models. Similarly, the values of $\log s_{\sigma_{0}}$ were almost identical for both models (Table 1). In contrast, the values of $s_{\mu_{0}}$ for Model 2 were smaller than those for Model 1.

Next, to compare two models, loo-ic and WAIC were calculated. The value of loo-ic is small for Model 1, whereas that of WAIC is small for Model 2 (Table 4). However, differences between Model 1 and Model 2 were very small (−0.5 in loo-ic and 0.7 in WAIC).

### 3.4. Probability Curve Fitting to Raw Data by Estimated Parameters

**Figure 3:**
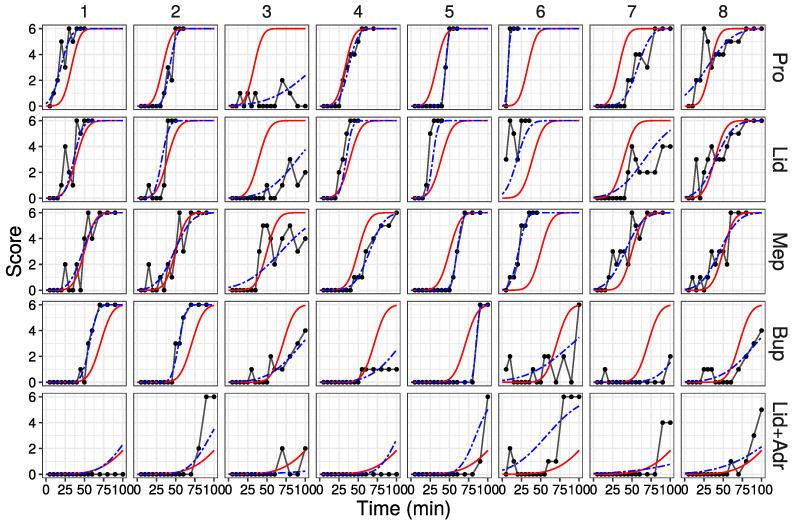
Fitted probability curves by parameters estimated in Model 2. Red solid line: probability curve by the fixed effect model; blue dashed line: probability curve by the random effect model. First eight data are shown (All data are shown in Figure S3).

The probability curve calculated by the parameters estimated in Model 2 are shown in Figure 3 (All data are shown in Figure S3). The red solid lines are curves calculated from the parameters of each drug (fixed effect model). The blue dashed lines are curves fitted to each drug and individual (random effect model). The random effect model fit the raw data well. However, poor fitting was observed when the raw data score value underwent large up-and-down fluctuations.

### 3.5. Computer Simulation Using Estimated Parameters

Based on the estimated parameters (except *d*) in Table 1, score values were determined for 100 individuals by computer simulation. Simulation parameters used are listed in parameter 1 of Table 5. For the simulation using parameter 1, the SD of the offset values, *d*, was set to a smaller value than estimated, to reduce differences among individuals.

**Figure 4:**
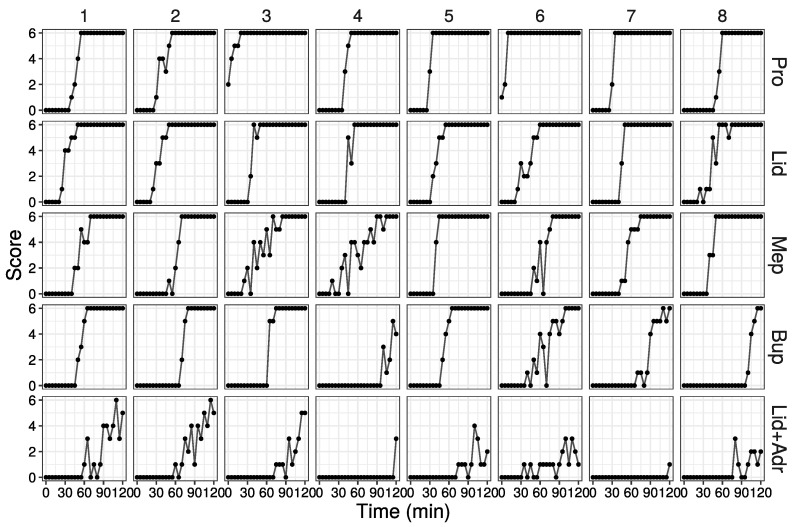
Results of simulation using estimated parameters (Parameter 1 in Table 5). First eight data out of 100 are shown (All data are shown in Figure S4).

Some simulation results are shown in Figure 4 (all data are shown in Figure S4). Over time, the increase in score values with up-and-down fluctuations was observed for all drugs and individuals. These results reflected the raw data. However, these fluctuations tended to continue when the slope of the curve was small, particularly in Lid + Adr.

### 3.6. Comparison of Local Anesthetic Agent Duration between Raw and Simulated Data

**Figure 5:**
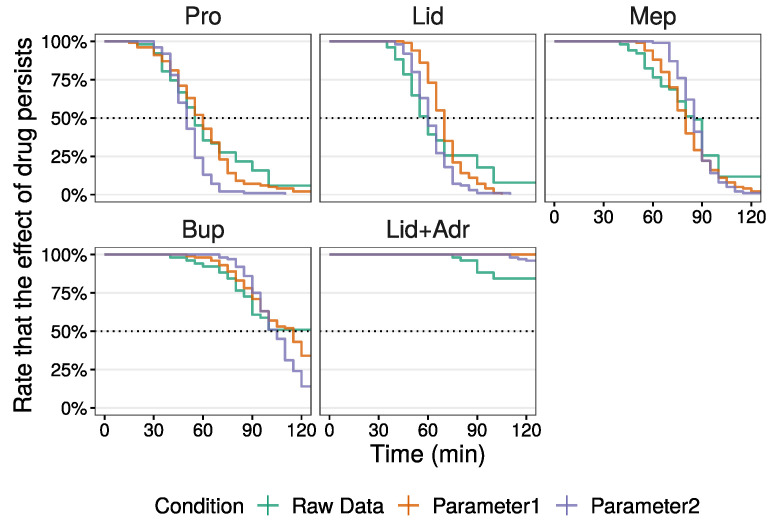
Kaplan–Meier curve of duration time in raw data and simulated data (parameters are listed in Table 5).

Next, the median of local anesthetic agent duration from the raw data and that simulated using parameter 1 were compared by survival analysis (Figure 5 and Table 6). Kaplan–Meier curves of the simulation data using parameter 1 (orange line) resembled that of the raw data (green line) except for Lid. Although the Kaplan–Meier curve for the Lid raw data initially decreased, this tapered off at a later stage. This curve from parameter 1 decreased in an inverted S shape. In contrast, though the effect of Lid + Adr disappeared in several individuals, for all individuals, using parameter 1, the effect was sustained. The median values for Pro and Lid using parameter 1 were large compared to those observed for the raw data. Moreover, the 95% confidence intervals (CI) in parameter 1 were narrow compared to those for the raw data. In this study, as the median value for Bup was uncalculatable for the raw data, a comparison of Bup duration between two conditions was not made. However, the Kaplan–Meier curves did show similarities.

Lastly, to approximate the raw data median values by simulation, several parameters were adjusted through trial and error (parameter 2 in Table 5). To change local anesthetic agent duration time, values of $\mu_{0}$ were adjusted. To decrease the spread in duration among individuals, the values of $s_{\mu_{0}}$ and $\log s_{\sigma_{0}}$, involved in the random effect model, were reduced compared to parameter 1 except for $\mu_{0}$ of Lid. The simulation results are shown in Figure S5. The Kaplan–Meier curves of simulation using parameter 2 (violet line) indicate a small spread in duration among individuals compared to the raw data and simulated data using parameter 1 (Figure 5). Median values of duration were almost identical to the raw data, and the 95% CI for parameter 2 were also narrow compared to those in the raw data (Table 6).

## 4. Discussion

In this study, a model was proposed with parameters estimated using the Bayesian hierarchical model and Hamiltonian Monte Monte method. Probability curves fitting each drug and individual data were obtained using these parameters. Moreover, when score values were predicted based on the estimated parameters with slight adjustments, these score values reflected data obtained by animal experiments.

### 4.1. Comparison with Other Studies Using Bayesian Hierarchical Models

As described in the Introduction, Bayesian hierarchical models are used in various areas. There are also expriments that perform modeling using the binomial distribution. Binomial distribution is generally used when the objective variable is binary data, similar to logistic regression analysis. Moreover, the binomial distribution is also used when the objective variable is count data. Examples of the latter include reports on its application to the analysis of the number of bacterial infections [26] and population growth curves [27]. These papers predict the probabilities or proportions, not actual numbers. On the other hand, there is a report to predict the spatial and temporal spread of infectious diseases in terms of the number of people, but results of point estimation and interval estimation were presented [28]. The statistical model in this study also uses the binomial distribution to estimate parameter values and predict probabilities. However, this study differs from other reports in that it calculates scores for each individual and uses random numbers.

### 4.2. About the Relation between Local Anesthetic Agents and Parameters

As described in the Introduction, the duration of local anesthetic agent is determined by peripheral vasodilation and lipid solubility [6,7]. Pro and Lid both induce peripheral vasodilation, Mep induces less vasodilation [6], and Bup induces vasoconstriction [6,7]. In addition, the lipid solubility of Bup is high. These characteristics rationalize the short durations of Pro and Lid, the relatively longer duration of Mep, and the very long duration of Bup. In these animal experiments, the local anesthetic agent duration order was as expected (Table 6). Also, $\mu_{0}$ values of local anesthetic drugs are also estimated in the correct order (Table 1). These results suggest that this model correctly estimates local anesthetic agent duration.

### 4.3. About the Models in This Study

#### 4.3.1. About Estimated Parameters

The estimated values of $\mu_{0}$ and $\sigma_{0}$ (in Table 1) and μ and σ (in Table 2 and Table 3) are similar for both Model 1 and 2. This suggests a similar degree of parameter estimation in both models. Models for which the estimates of loo-ic and WAIC are small are thought to be well fitting. In this study, both the estimates of loo-ic and WAIC were very close (Table 4), with small and negligible differences, suggesting that both models have the potential to better fit the raw data to the same degree.

Next, considering the difference of $s_{\mu_{0}}$ between both models. The $s_{\mu_{0}}$ values were small compared to those in Model 1 (Table 1). For Model 1, the individual difference is ignored. In contrast, for Model 2, individual difference is incorporated as the offset value *d* (Equation ()). As the dispersion of μ in Model 1 is divided between *d* and μ in Model 2, the dispersion of μ in Model 2 becomes smaller. As a result, the $s_{\mu_{0}}$ values in Model 2 are considered small compared to those of Model 1. These results imply that Model 2 is preferable, despite both models fitting to the same degree.

The small $s_{\mu_{0}}$ value has some advantage when the score values are obtained in the computer simulation. As $\mu_{0}$ values of Pro and Lid are relatively close, it is possible that μ values of Pro and Lid randomly generated in the computer simulation are distinct to the proper order of $\mu_{0}$. As a result, the duration of Pro and Lid will sometimes reverse. When the $s_{\mu_{0}}$ values are small, the probability that the μ values and resulting duration among local anesthetic agents reverse (in particular between Pro and Lid) is small. In the computer simulation experiments, the probabilities that the μ values of Pro and Lid reverse were approximately 40% in Model 1 and 36% in Model 2. Moreover, after parameters were adjusted (parameter 2 in Table 5), this probability is decreased to 20%. Thus, further parameter adjustments are possible.

#### 4.3.2. About Overfitting in This Model

Statistical models are typically created to predict results obtained in new individuals or conditions. To evaluate statistical models, external validity is essential. For this purpose, all data are usually analyzed by dividing them into model creation and model verification. In contrast, because all data were used for model creation, our model cannot be verified for the external validity and may overfit data from animal experiments. However, the primary purpose of this study is to obtain the results close to animal experiments and with less variation in a simulator. Considering this purpose, we think that this overfitting may be less problematic.

### 4.4. About Usefulness of This Model

For the animal experiment raw data, the dispersion in score values was large due to technical errors such as failure of injection and inconsistent stimulus intensity, resulting in large variations in estimated parameters ($s_{\mu_{0}}$) (Table 1). In contrast, by setting $s_{\mu_{0}}$ values small in the computer simulation (parameter 2 in Table 6), the dispersion of score values was small, and 95% CI was narrow. Moreover, these technical errors were avoided in the computer simulation. These results suggest that this model is effective at local anesthetic agent effect simulation, to the point that unexpected results are unlikely to be obtained.

For the computer simulation of this model, it facilitates learning about (1) duration comparisons among several local anesthetic agents, and (2) the interaction with adrenaline as a vasoconstrictor in terms of duration as well as in the method using animals. Therefore, this model presents an alternative to animal use in the practice of local anesthetic agents. We consider that the replacement of animals is possible, and is an essential objective in conducting humane science.

### 4.5. Limitation of This Model

There are several limitations in this model, as follows.

#### 4.5.1. Non-Randomized Experimental Design

In this study, randomization was not considered. The reason is that the purpose of the practice is to confirm and understand the known effects of local anesthetic agents. Indeed, randomization is necessary in quantitative experiments. However, this practice is a qualitative experiment that compares the action duration of several local anesthetic agents, and the expected results are actually obtained in this simulation.

#### 4.5.2. Remarkable Up-and-down Fluctuations of Effects

The up-and-down fluctuations of effects in simulation disadvantage this model. In animal experiments, different responses occur depending on several factors such as (1) differences in drug concentration within the area where local anesthetics are administered (high concentration in the center, and low concentration in the periphery), and (2) variations in the strength of stimulation. In order to reflect this variation in responses in the simulation, our statistical model calculated the probability of responding to a stimulus and used random numbers that follow a binomial distribution based on that probability. As random numbers are not affected by the previous value, the obtained score values fluctuate up and down. In fact, the up-and-down score value fluctuations were observed in all drugs and individuals (Figure 4). In particular, the up-and-down fluctuations were clear for large σ values (including the effect of adrenaline). However, when the probability of response is close to 0 or 1, up-and-down fluctuations are unlikely to occur. Therefore, we consider that this up-and-down fluctuation is less problematic when examining the duration of local anesthetics agents in simulations.

It is possible to reduce the up-and-down fluctuation range by increasing the number of stimulations. The value of the SD for binomial distributions is determined by the number of trials and probability. As the number of reaction-inducing stimulations (*n* = 6) in this study is small, the value of SD is large, and therefore the stochastic variation is unavoidable. Larger numbers of stimulations will decrease the value of SD and therefore diminish the up-and-down fluctuations in the simulation. However, in the case of many stimulations, the conditions for the subsiding local anesthetic agent will change. Thus, further examination is required for simulation.

#### 4.5.3. Insufficient Response to Simulation Immediately after Administration

Moreover, this model assumes an exponential decrease in local anesthetic agent concentration (i.e., the logarithm concentration decreases linearly). Therefore, the course from administration to the onset of action was not considered, assuming an immediate local anesthetic agent effect after administration. However, this is considered less problematic as the primary purpose of this experiment (as well as the animal experiments) is to compare duration among drugs.

#### 4.5.4. Difficulty Dealing with NEW Conditions

For the same reason that extrapolation in regression analysis is inappropriate, this model is disadvantaged for new drugs. As parameter values were estimated using the results in practice, this model simulates only known drugs with concentration data. In the case of new drugs, or new concentrations or doses, it is unable to simulate these situations. In these cases, animal experiments are necessary to estimate parameters for the said new conditions.

## 5. Conclusions

In this study, we assumed a statistical model and estimated the parameters of each local anesthetic agent. Score values predicted using new parameters randomly generated from the estimated parameter reflected that of the raw data obtained from animals. Benefits of this model include no animal usage, no technical failure, and small dispersions in score values. The statistical model in this study provides a novel theoretical background to create a simulator for local anesthetic agents. It therefore becomes possible to create the simulator using parameter values estimated in this study.

## Figures and Tables

**Table 1:** Mean of parameters estimated from posterior distribution (fixed effect model: $\mu_{0}\left[i\right]$ and $\sigma_{0}\left[i\right]$; random effect model: $s_{\mu_{0}}\left[i\right]$ and $\log s_{\sigma_{0}}\left[i\right]$).

		Model 1		Model 2	
i	Drug	$\mu_{0}\;\left(s_{\mu_{0}}\right)$	$\sigma_{0}\;\left[\log\sigma_{0},\log s_{\sigma_{0}}\right]$	$\mu_{0}\;\left(s_{\mu_{0}}\right)$	$\sigma_{0}\;\left[\log\sigma_{0},\log s_{\sigma_{0}}\right]$
1	Procaine	67.5 (19.5)	9.0 [2.19, 0.79]	67.0 (15.7)	9.0 [2.20, 0.78]
2	Lidocaine	61.0 (14.8)	11.3 [2.43, 0.52]	60.9 (5.1)	11.3 [2.42, 0.51]
3	Mepivacaine	50.5 (14.5)	11.1 [2.41, 0.65]	50.0 (12.0)	11.1 [2.41, 0.65]
4	Bupivacaine	29.3 (26.0)	12.1 [2.50, 0.83]	29.1 (20.3)	12.2 [2.50, 0.83]
	Adrenaline	0.663		0.665	
	offset value (d)			0.35 (13.0)	

**Table 2:** Mean of estimated parameters for each animal in Model 1. First ten data out of 51 are shown as μ[i,j](σ[i,j]).

Animal (*j*)	Procaine	Lidocaine	Mepivacaine	Bupivacaine
1	80.9 (10.5)	63.6 (9.2)	52.0 (13.9)	43.3 (8.0)
2	57.7 (6.7)	68.1 (7.3)	51.5 (18.3)	46.1 (7.2)
3	−5.4 (41.7)	11.3 (31.8)	39.8 (32.8)	10.4 (30.4)
4	62.4 (9.8)	66.1 (6.3)	34.8 (15.8)	−9.5 (32.7)
5	55.1 (3.3)	71.8 (5.4)	39.9 (5.9)	15.1 (3.5)
6	92.6 (2.5)	80.4 (12.2)	78.8 (8.4)	1.9 (50.8)
7	43.1 (13.8)	32.1 (29.8)	60.7 (18.3)	−13.6 (21.9)
8	71.1 (26.9)	59.6 (18.4)	54.8 (19.2)	5.1 (35.9)
9	103.9 (28.5)	31.1 (14.9)	11.1 (6.4)	−20.2 (8.1)
10	50.0 (13.0)	59.0 (10.7)	47.4 (10.8)	25.5 (14.0)

**Table 3:** Mean of estimated parameters for each animal in Model 2. First ten data out of 51 are shown as μ[i,j](σ[i,j]).

Animal (*j*)	Procaine	Lidocaine	Mepivacaine	Bupivacaine
1	80.8 (10.4)	63.9 (9.2)	52.2 (13.9)	43.2 (8.1)
2	57.8 (6.8)	68.1 (7.4)	51.8 (18.2)	46.0 (7.2)
3	−11.9 (44.9)	10.3 (32.2)	31.5 (37.8)	4.2 (34.3)
4	62.4 (9.7)	65.8 (6.3)	35.1 (15.8)	−6.4 (31.1)
5	55.1 (3.3)	71.8 (5.4)	40.1 (5.8)	15.2 (3.5)
6	92.6 (2.5)	80.6 (12.0)	79.0 (8.4)	8.8 (45.7)
7	42.6 (13.9)	33.6 (29.1)	59.0 (18.6)	−15.2 (23.3)
8	71.0 (26.8)	59.7 (18.4)	54.6 (19.4)	7.2 (34.7)
9	97.0 (24.0)	30.9 (14.9)	10.5 (6.4)	−22.2 (8.6)
10	50.2 (13.0)	58.8 (10.9)	47.2 (10.9)	25.6 (13.9)

**Table 4:** Leave-one-out cross validation information criterion (loo-ic) and widely applicable information criterion (WAIC) for Model 1 and Model 2.

Model	loo-ic		WAIC	
	Estimate	SE	Estimate	SE
Model 1	7113.7	181.1	7038.7	178.6
Model 2	7114.2	182.1	7037.4	178.9

**Table 5:** Parameters used in the computer simulations.

	Parameter 1		Parameter 2	
Drug	$\mu_{0}\;\left(s_{\mu_{0}}\right)$	$\sigma_{0}\;\left[\log\sigma_{0},\log s_{\sigma_{0}}\right]$	$\mu_{0}\;\left(s_{\mu_{0}}\right)$	$\sigma_{0}\;\left[\log\sigma_{0},\log s_{\sigma_{0}}\right]$
Procaine	67.0 (15.7)	9.0 [2.20, 0.78]	75.0 (8.0)	9.0 [2.20, 0.40]
Lidocaine	60.9 (5.1)	11.2 [2.42, 0.51]	67.0 (5.0)	11.0 [2.40, 0.40]
Mepivacaine	50.0 (12.0)	11.1 [2.41, 0.65]	43.0 (6.0)	11.0 [2.40, 0.40]
Bupivacaine	29.1 (20.3)	12.2 [2.50, 0.83]	30.0 (10.0)	12.2 [2.50, 0.50]
Adrenaline	0.665		0.700	
offset value (d)	0 (4.0)		0 (4.0)	

**Table 6:** Median of duration of local anesthesia under each condition.

Drug	Condition	*n*	Events	Median [95% CI]
Pro	Raw Data	51	48	55 [50, 65]
	Parameter 1	100	98	60 [55, 65]
	Parameter 2	100	100	50 [50, 55]
Lid	Raw Data	51	47	60 [55, 70]
	Parameter 1	100	100	70 [65, 70]
	Parameter 2	100	100	60 [60, 65]
Mep	Raw Data	51	45	85 [75, 90]
	Parameter 1	100	98	80 [75, 85]
	Parameter 2	100	99	85 [85, 90]
Bup	Raw Data	51	25	– [90, –]
	Parameter 1	100	66	115 [100, 120]
	Parameter 2	100	86	105 [100, 110]
Lid+Adr	Raw Data	51	8	– [–, –]
	Parameter 1	100	0	– [–, –]
	Parameter 2	100	4	– [–, –]

## Data Availability

Data are contained within the article and Supplementary Materials.

## Supplementary Materials

The following supporting information can be downloaded at: https://www.mdpi.com/article/10.3390/medicines10110061/s1, Figure S1: Raw data from animal experiments in the practice of pharmacology; Figure S2: Posterior distributions of several parameters obtained by Model 1 and Model 2; Figure S3: Fitted probability curves by parameters estimated in Model 2; Figure S4: Results of simulation using estimated parameters (Parameter 1 in Table 5); Figure S5: Results of simulation using estimated parameters (Parameter 2 in Table 5); supplementary data: raw data and programs.

## Abbreviations

The following abbreviations are used in this manuscript:

Pro	Procaine
Lid	Lidocaine
Mep	Mepivacaine
Bup	Bupivacaine
Adr	Adrenaline
SD	standard deviation
