# Supplementary material for: Development of a Predictive Statistical Pharmacological Model for Local Anesthetic Agent Effects with Bayesian Hierarchical Model Parameter Estimation
Source: Medicines (Basel). 2023 Nov 15;10(11):61. doi: 10.3390/medicines10110061 (PMC10672774; doi:10.3390/medicines10110061)
Supplement: Supplementary file 1 [file medicines-10-00061-s001.zip › Supplemental_legend.pdf]

SFig. 1

Raw data from animal experiments in practice of pharmacology

SFig. 2

Posterior distributions of several parameters obtained by Model 1 and Model 2. (Left) trace plot, (Right) density plot. `mu0`, `log_sigma0`, and `adr` means μ_0, $\log \sigma_0$, and adrenaline respectively. Numbers in brackets mean as follows. 1: Procaile, 2: Lidocaine, 3: Mepivacaine, and 4: Bupivacaine.

SFig. 3

Fitted probability curves by parameters estimated in Model 2. Red solid line: probability curve by the fixed effect model, Blue dashed line: probability curve by the random effect model.

SFig. 4

Results of simulation using estimated parameters (Parameter 1 in Table 5)

SFig. 5

Results of simulation using estimated parameters (Parameter 2 in Table 5)
