# Supplementary figures and images for: Development of a Predictive Statistical Pharmacological Model for Local Anesthetic Agent Effects with Bayesian Hierarchical Model Parameter Estimation
Source: Medicines (Basel). 2023 Nov 15;10(11):61. doi: 10.3390/medicines10110061 (PMC10672774; doi:10.3390/medicines10110061)

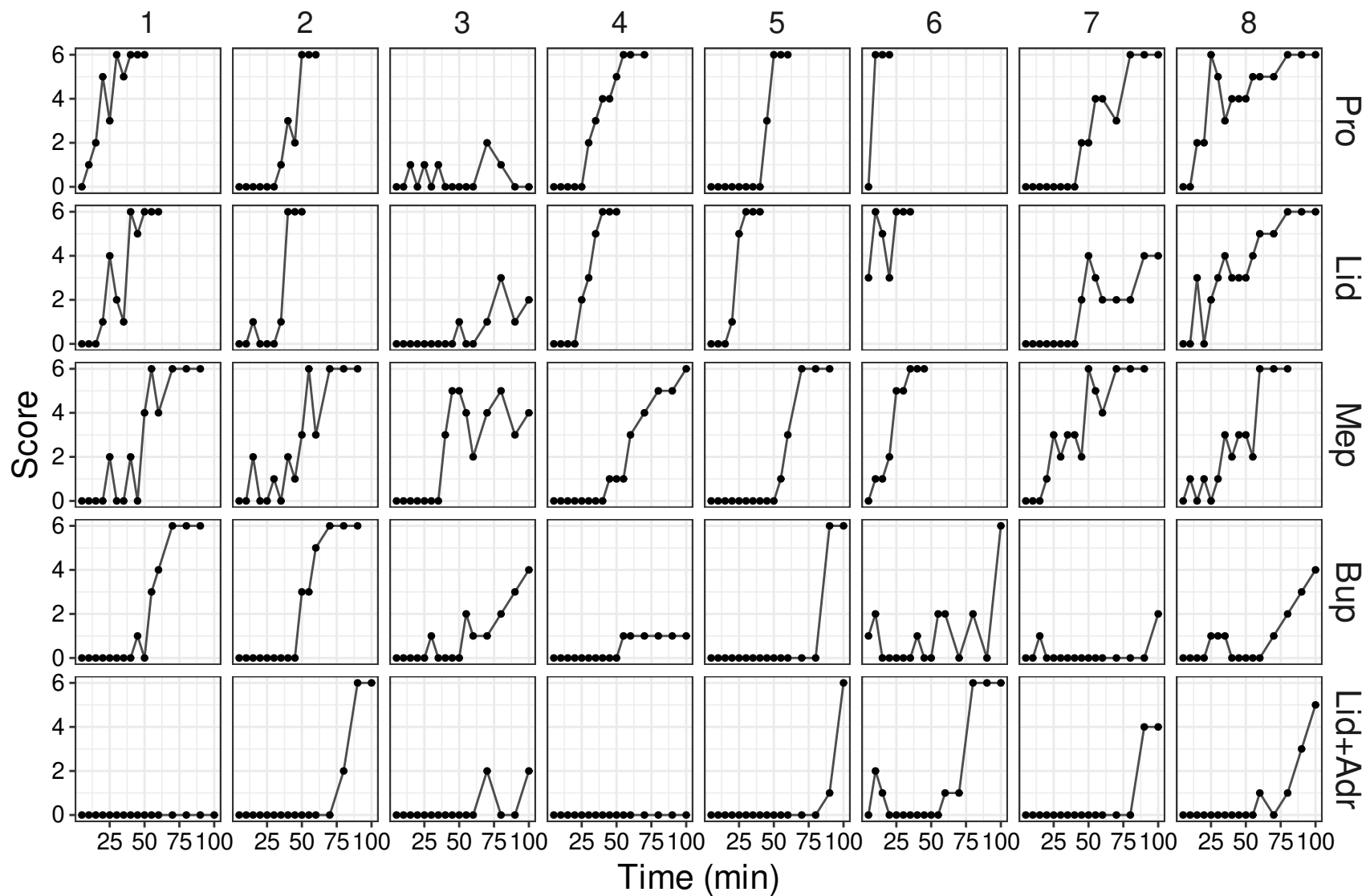

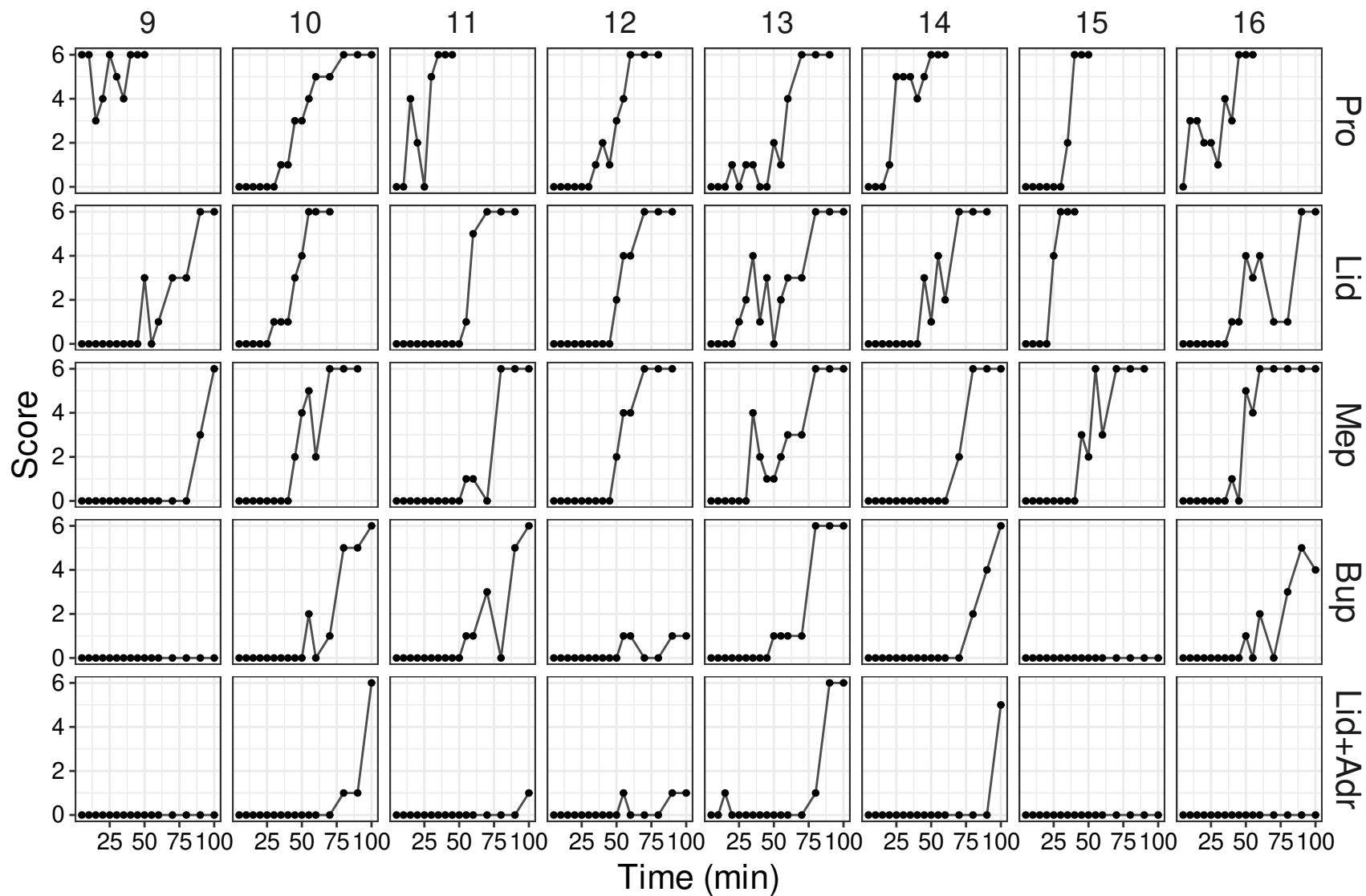

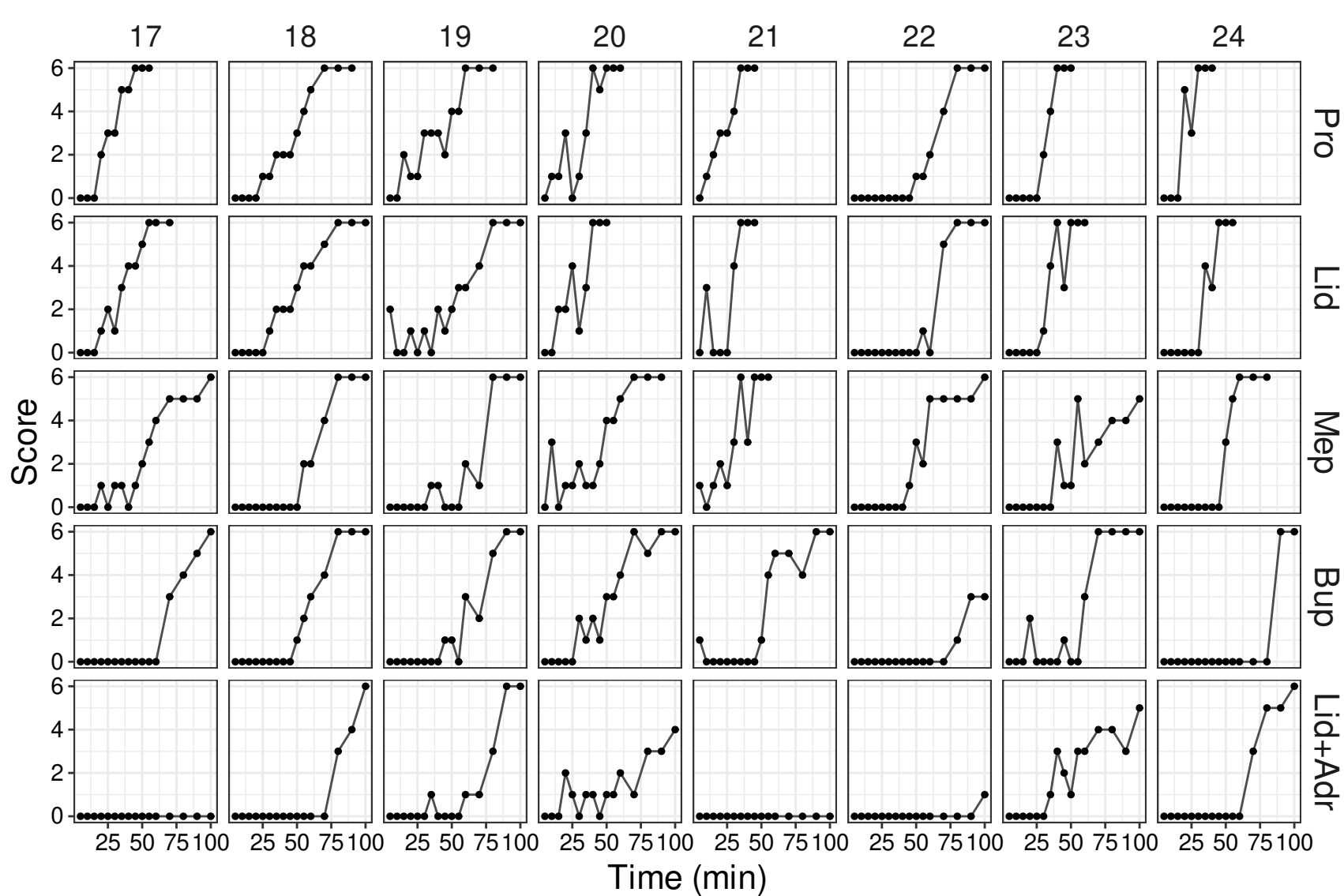

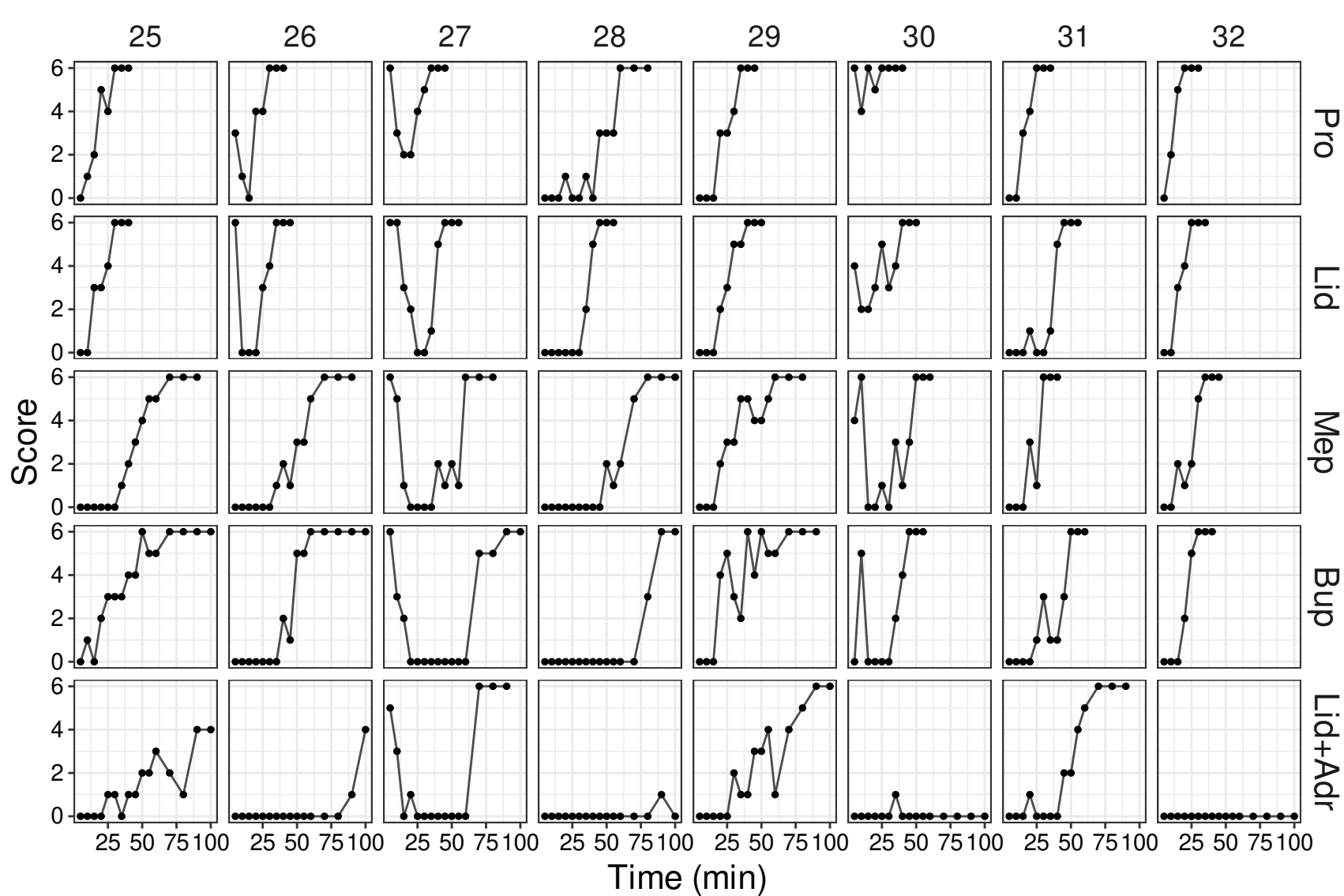

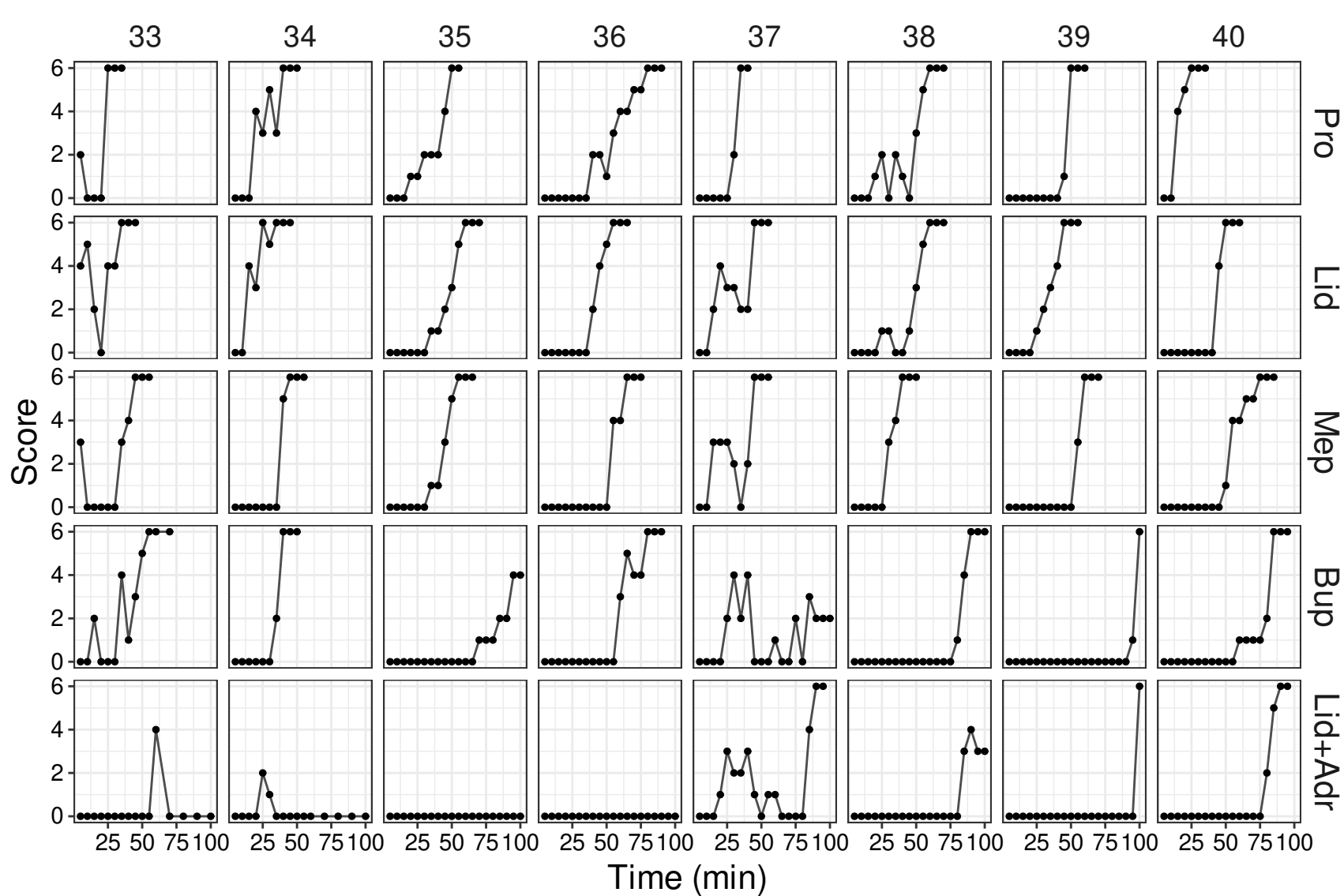

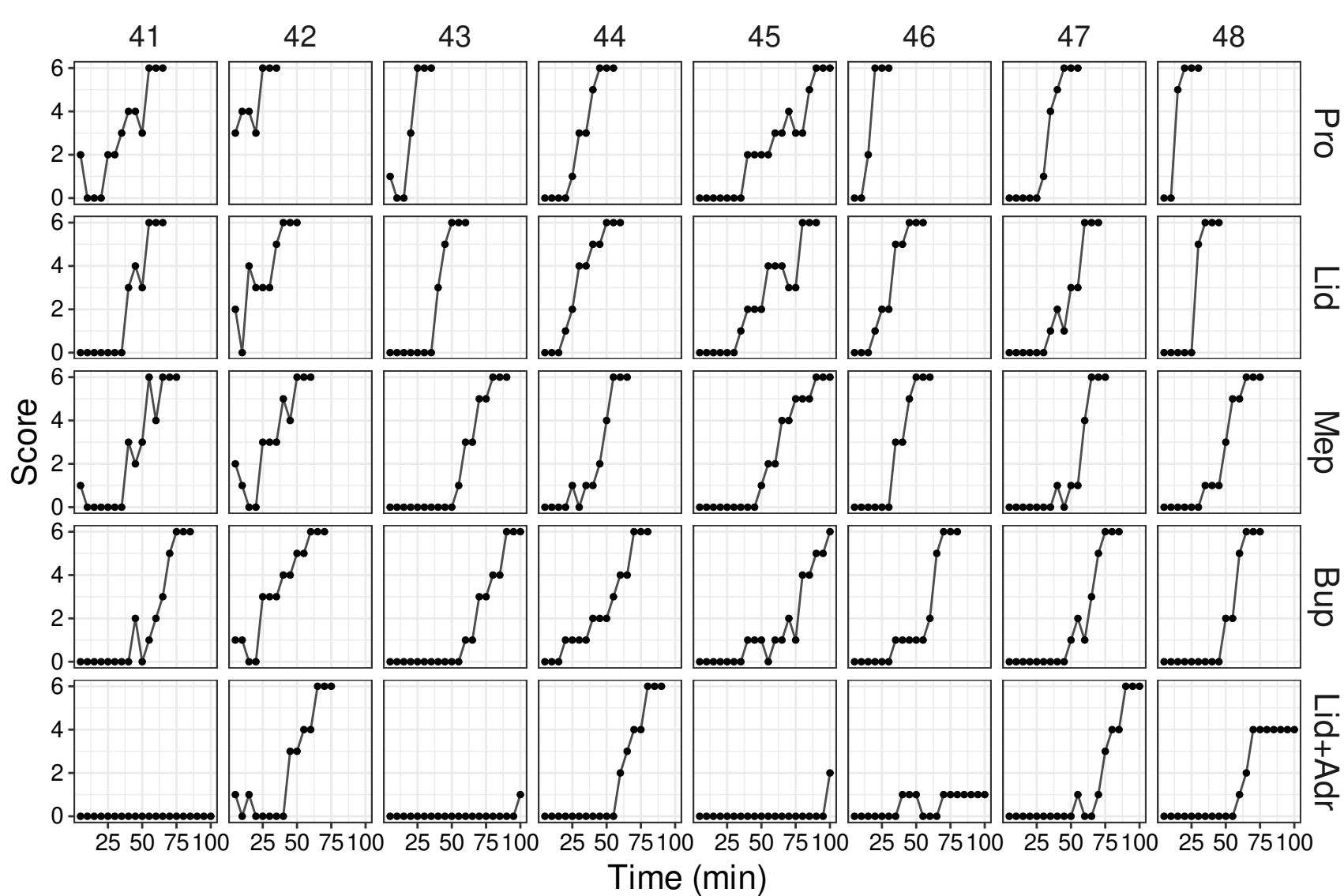

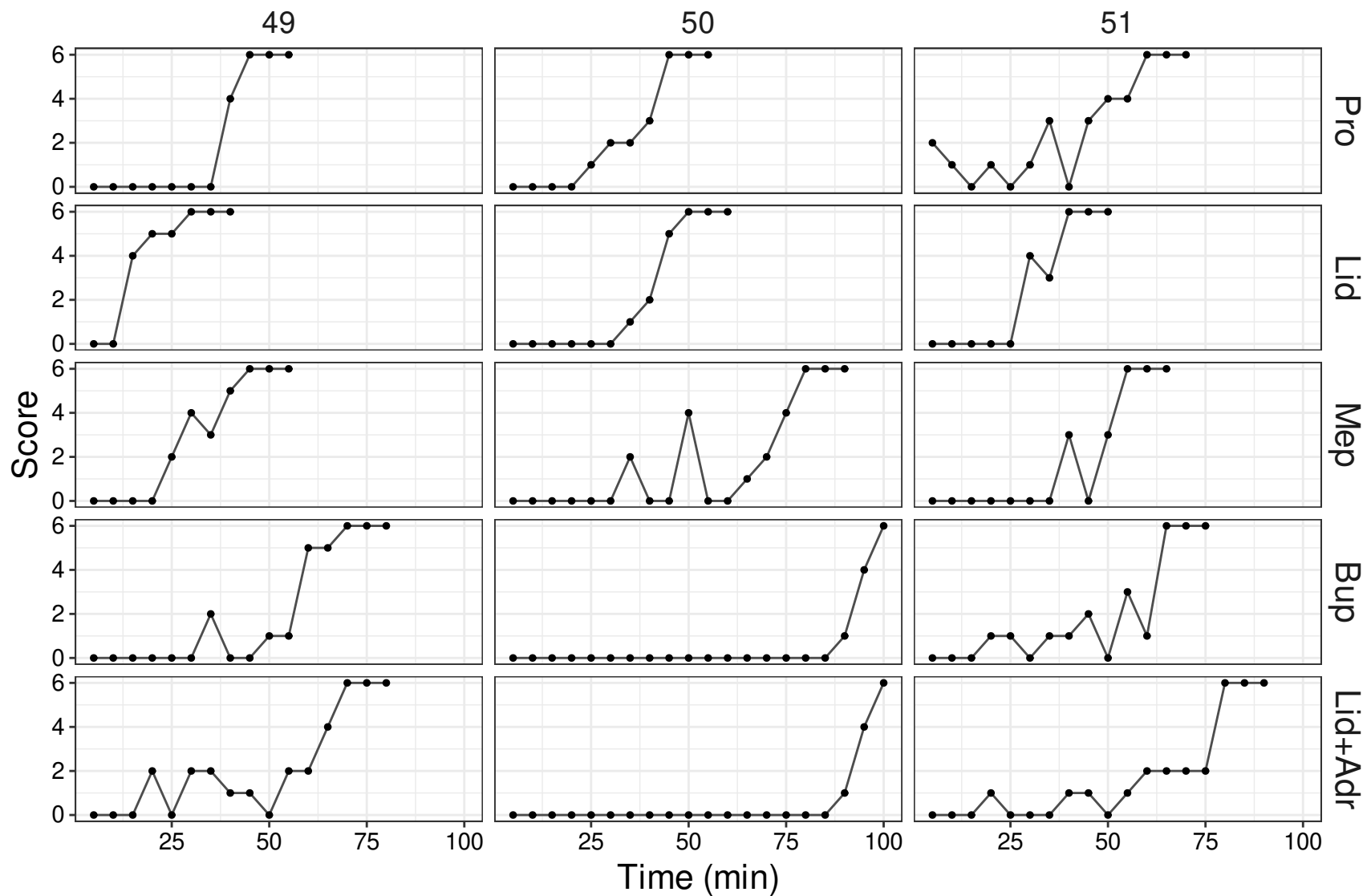

Supplement: Supplementary file 1 [file medicines-10-00061-s001.zip › SFig1.pdf]

# Model 1

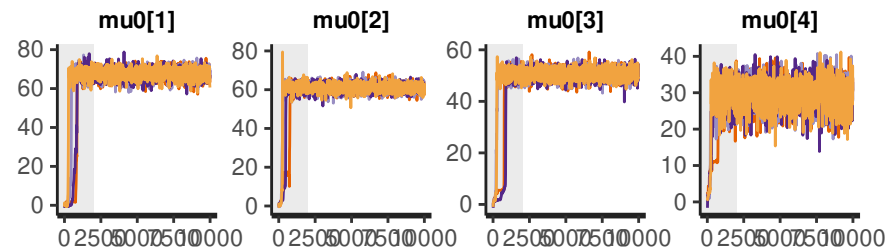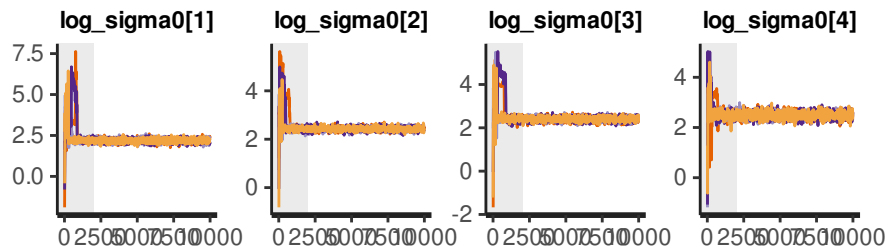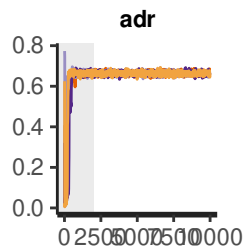

chain

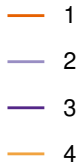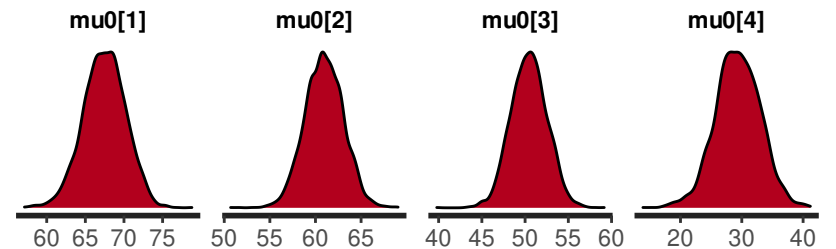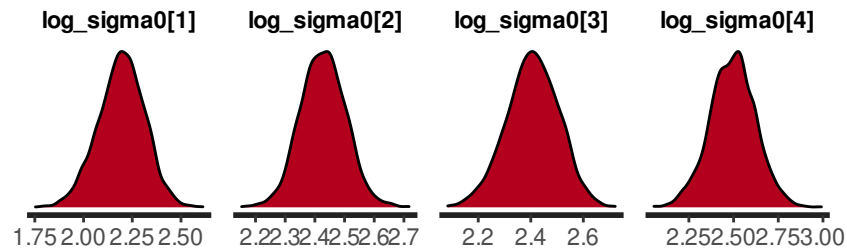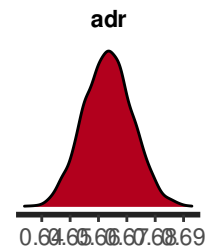

# Model 2

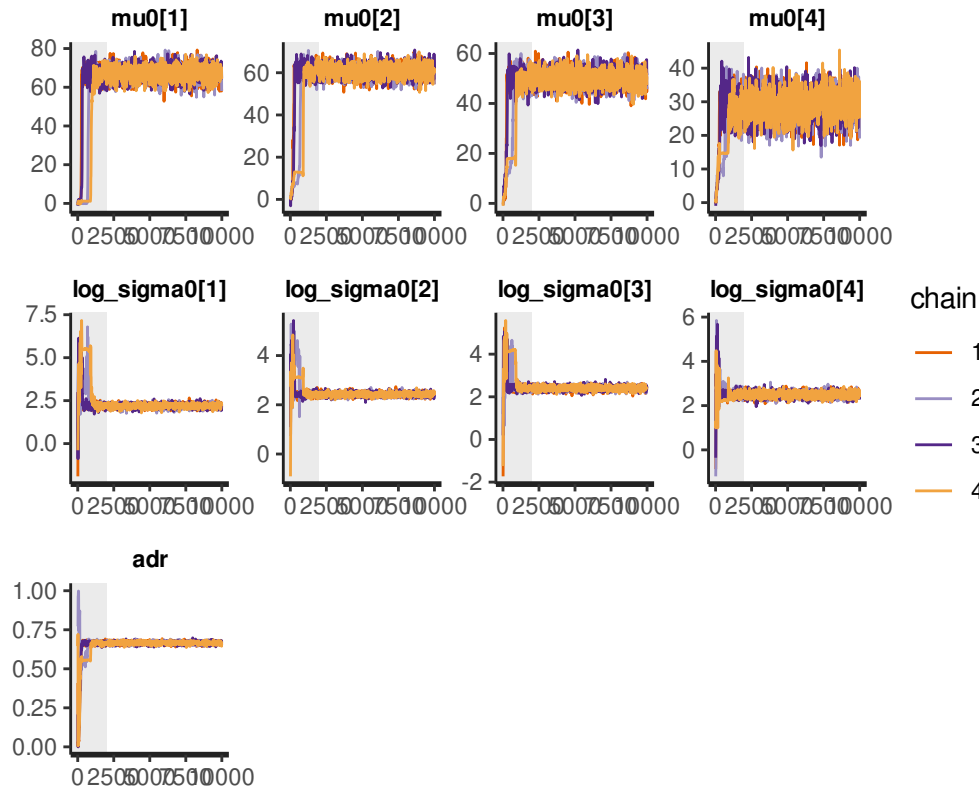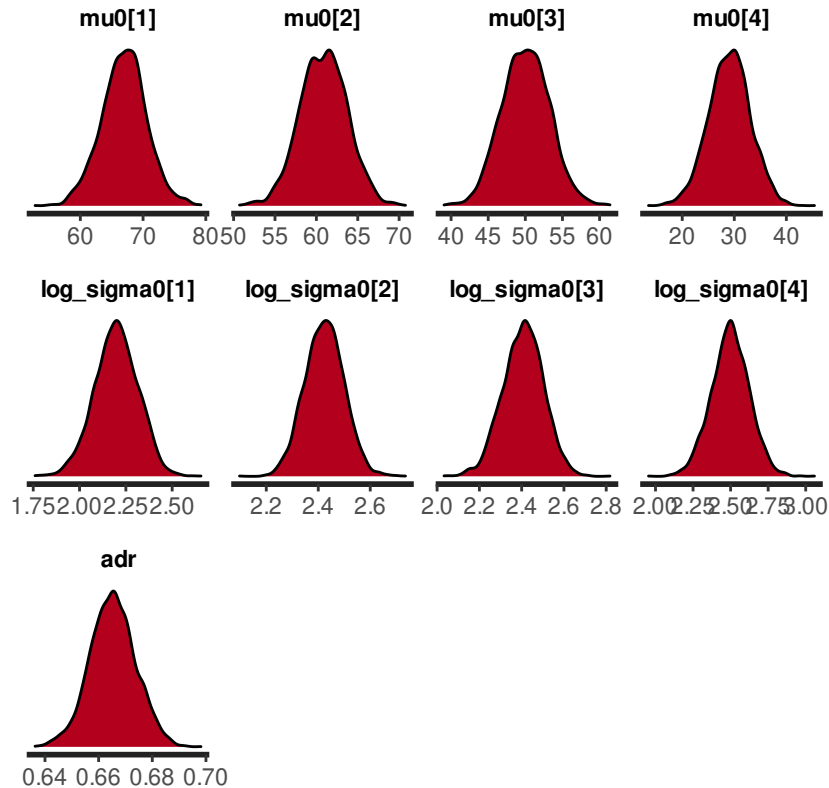

Supplement: Supplementary file 1 [file medicines-10-00061-s001.zip › SFig2.pdf]

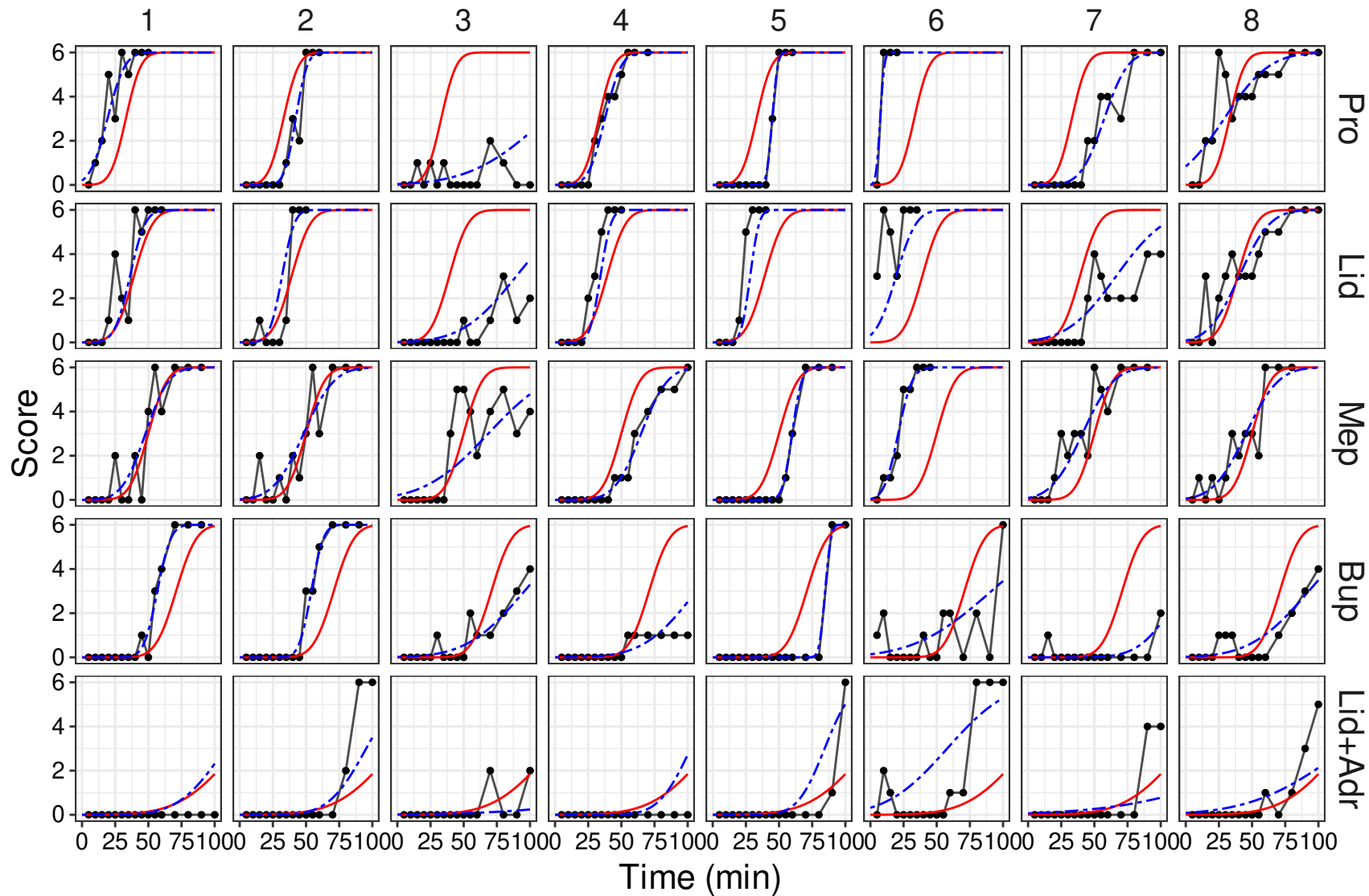

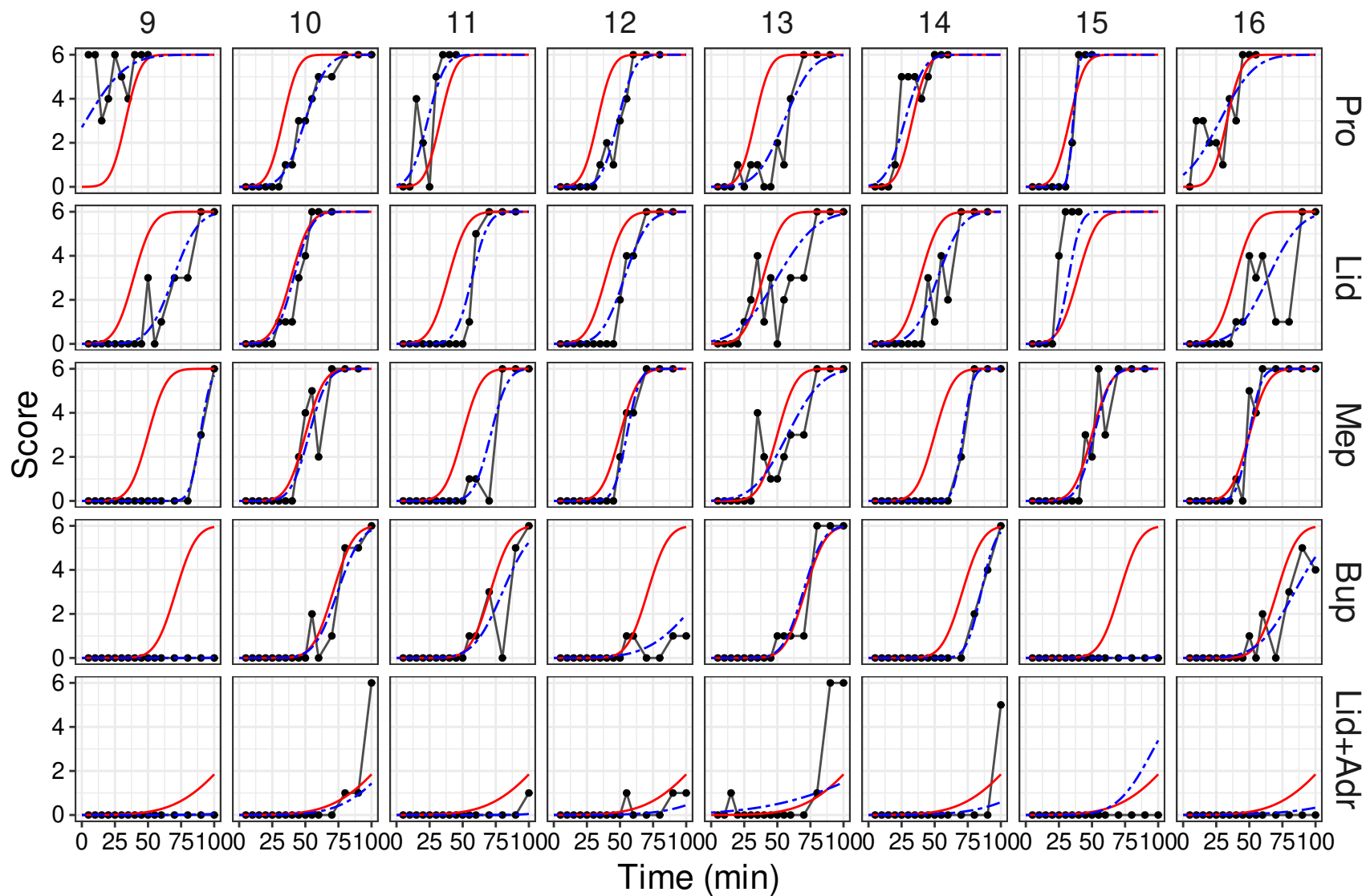

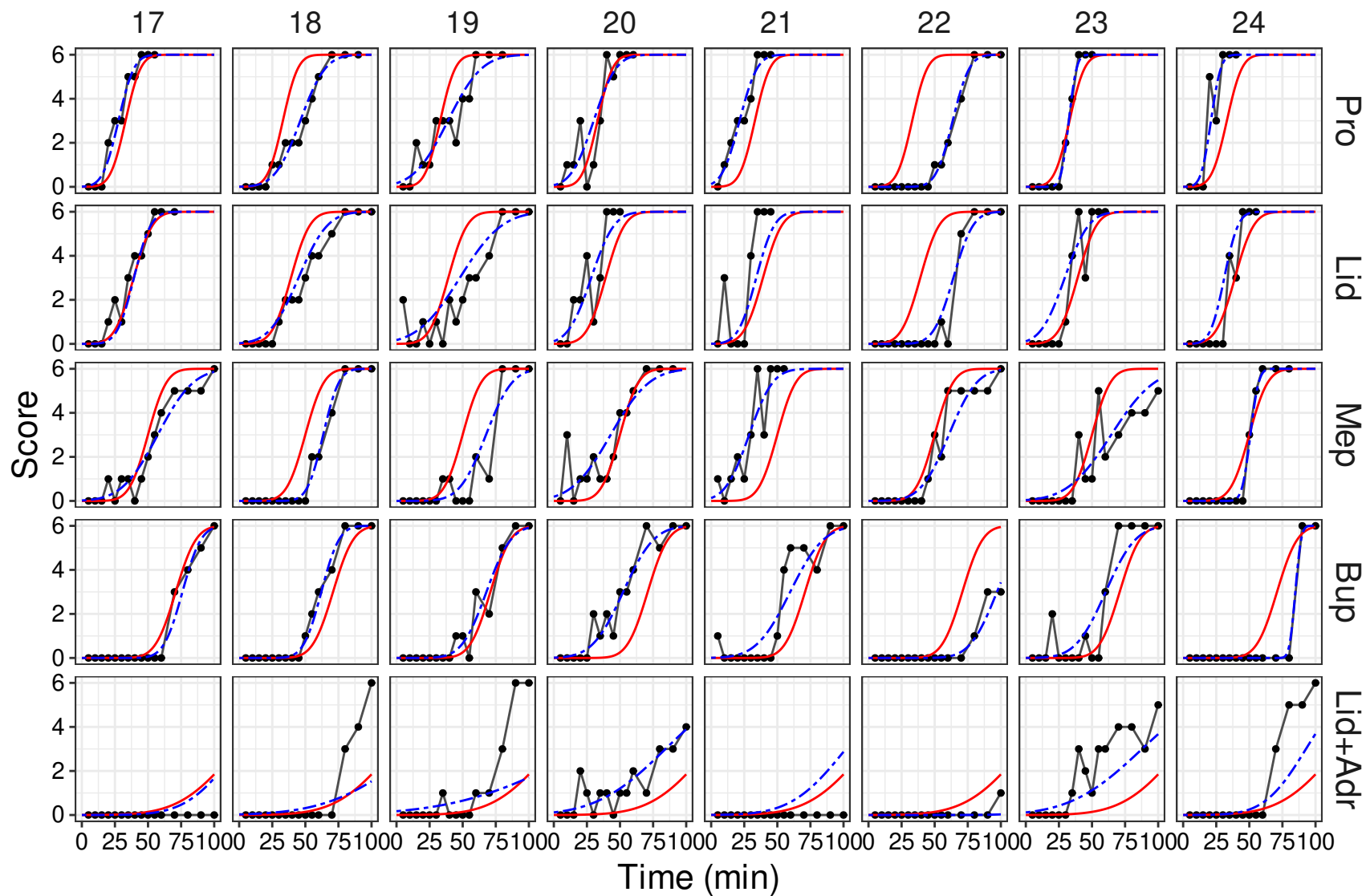

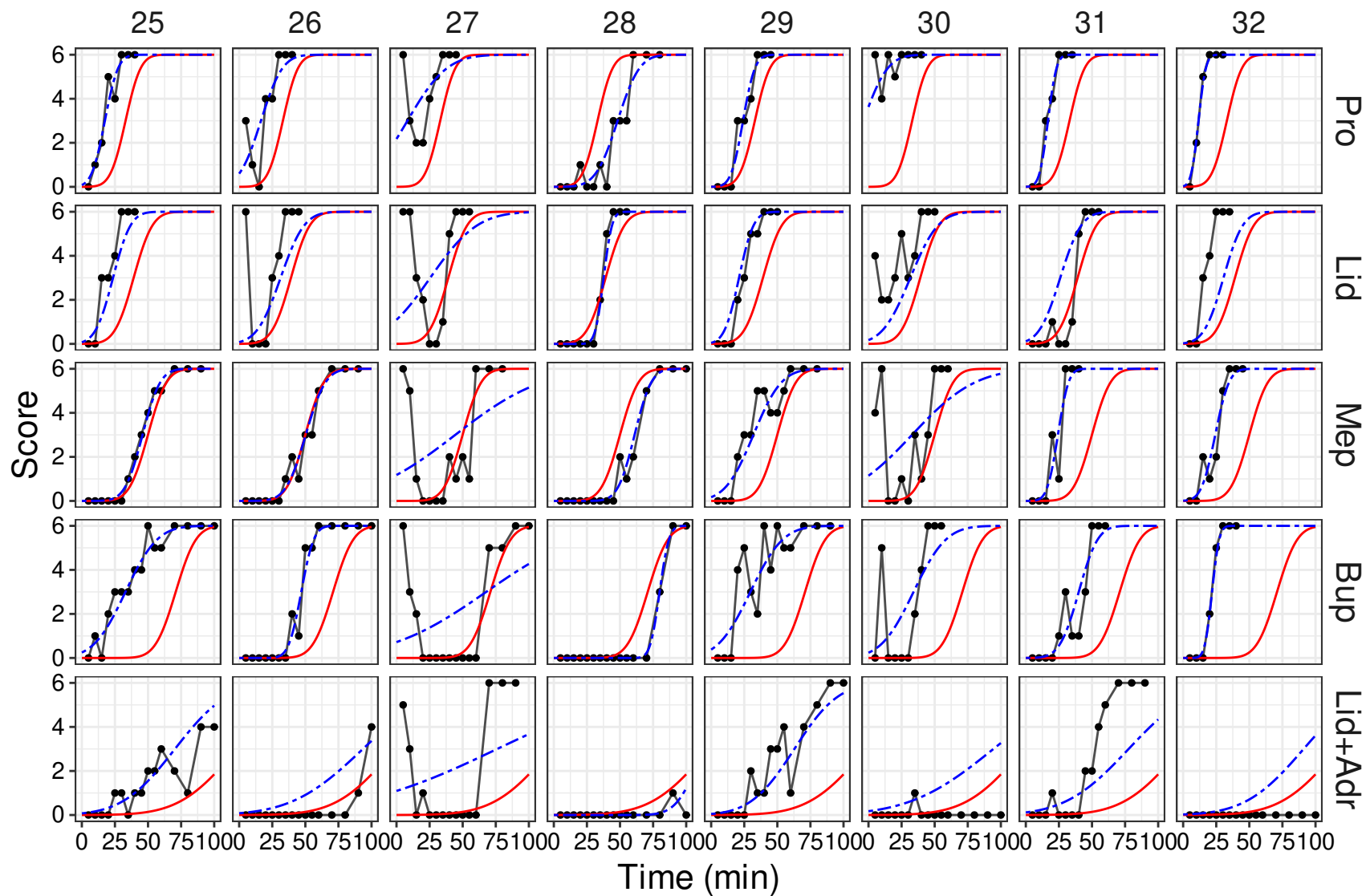

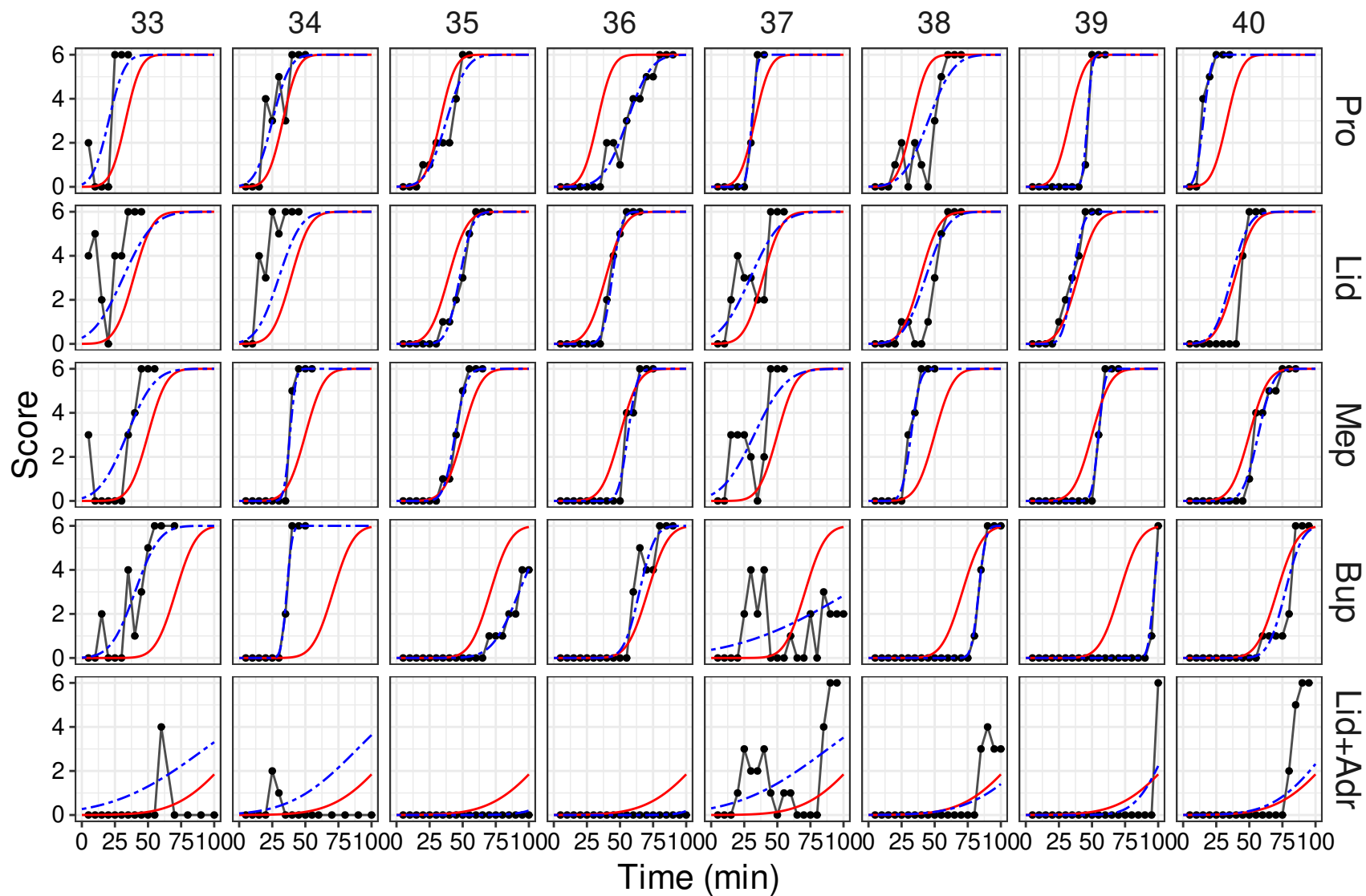

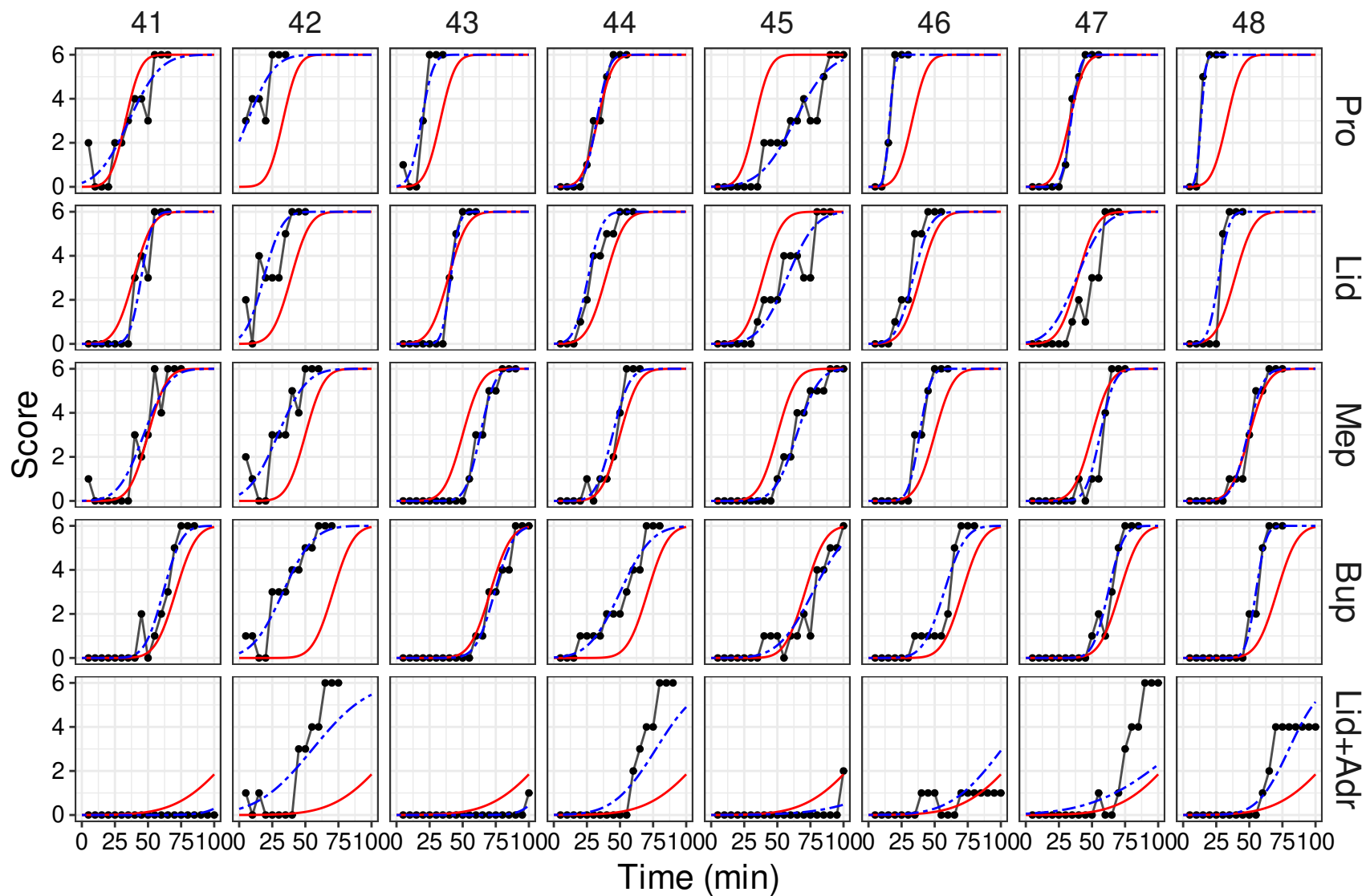

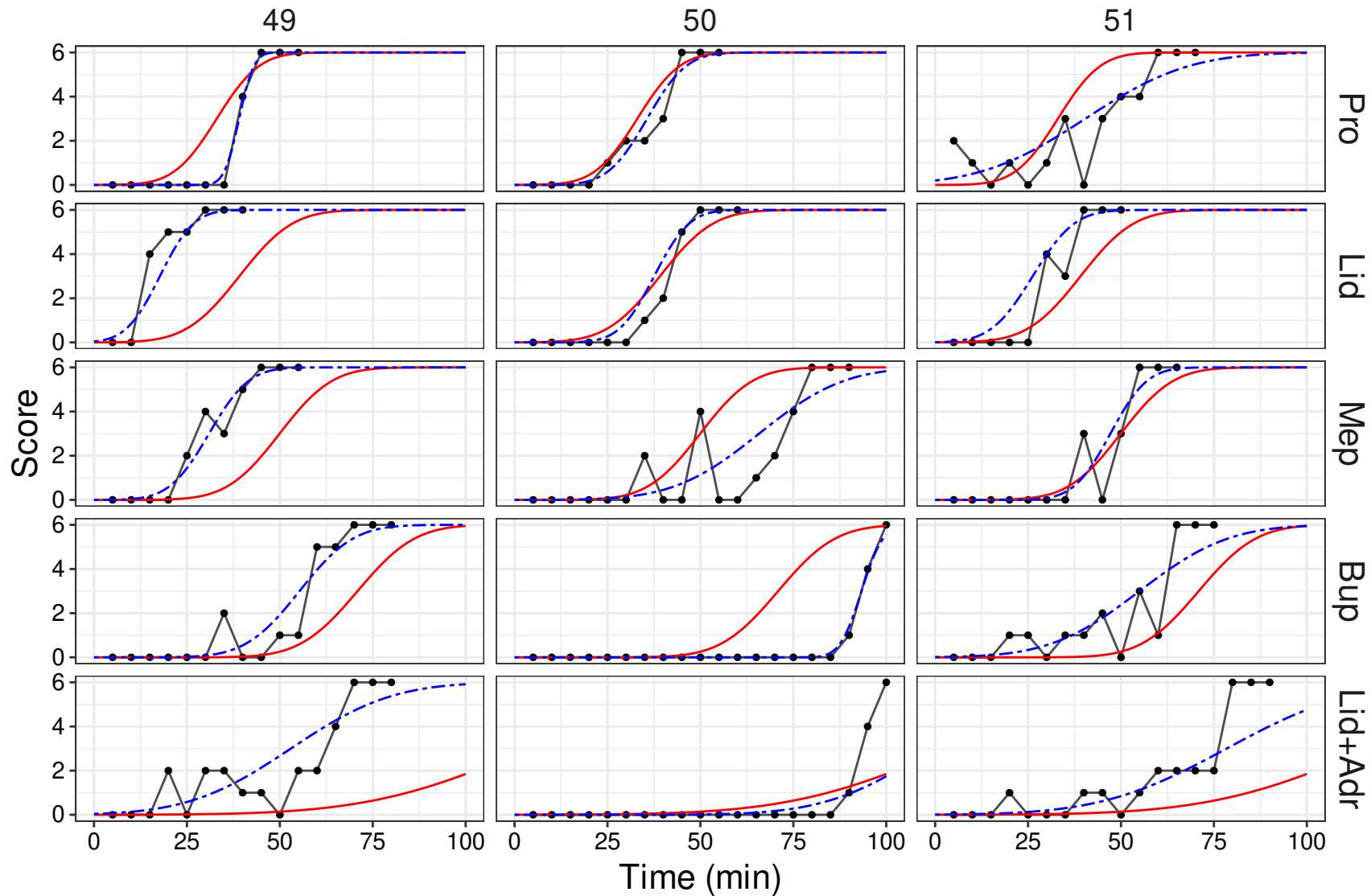

Supplement: Supplementary file 1 [file medicines-10-00061-s001.zip › SFig3.pdf]

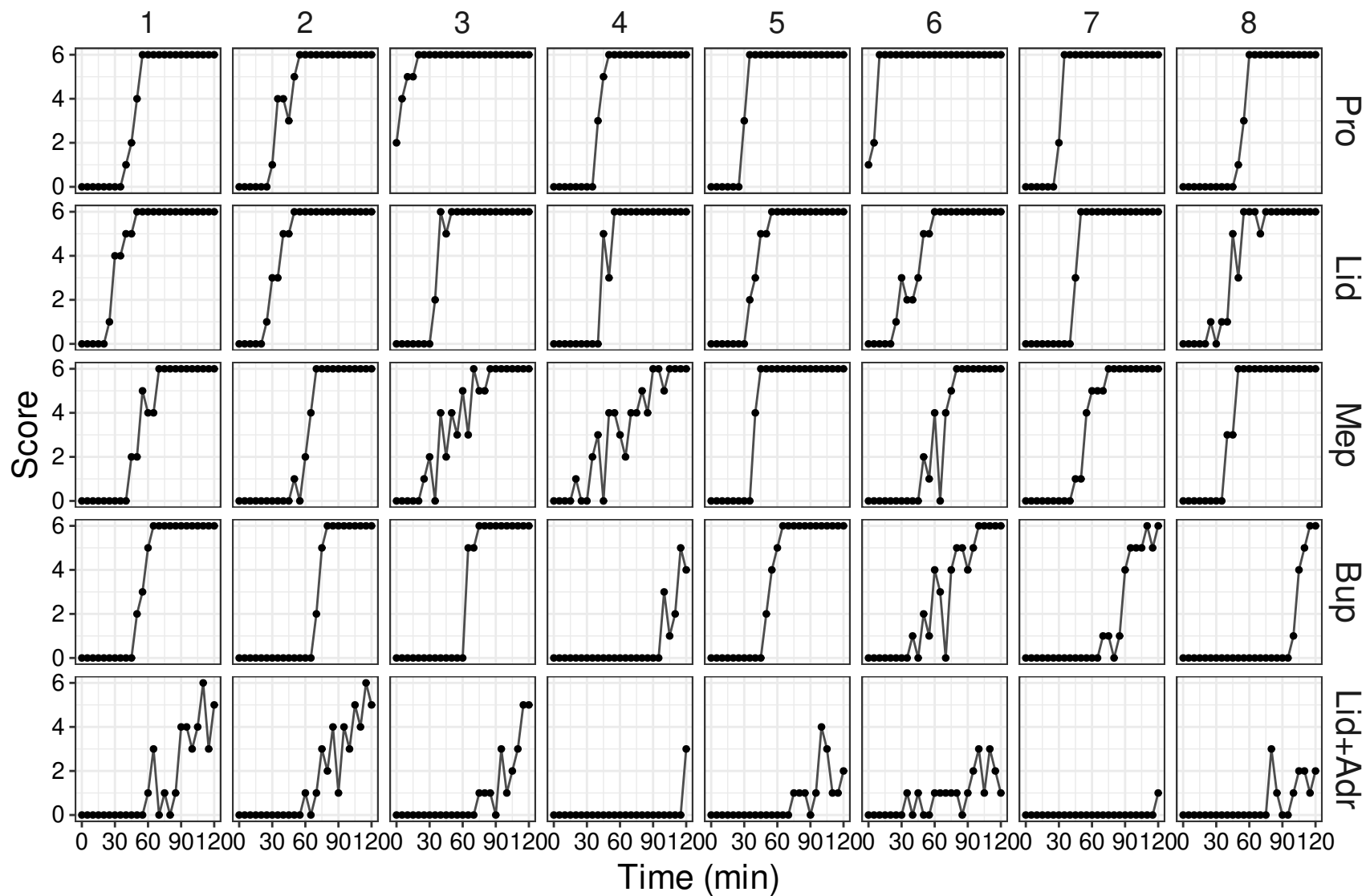

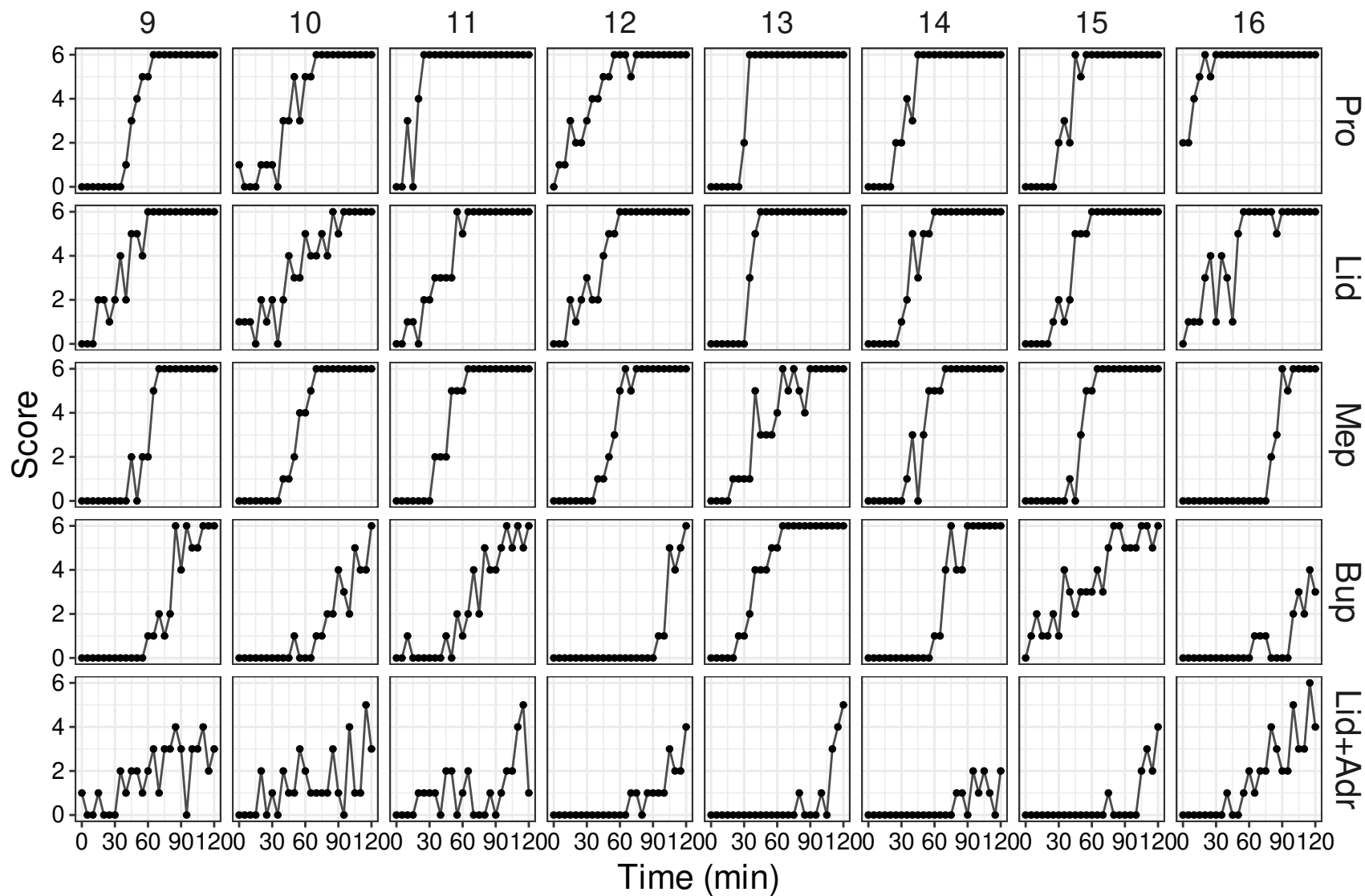

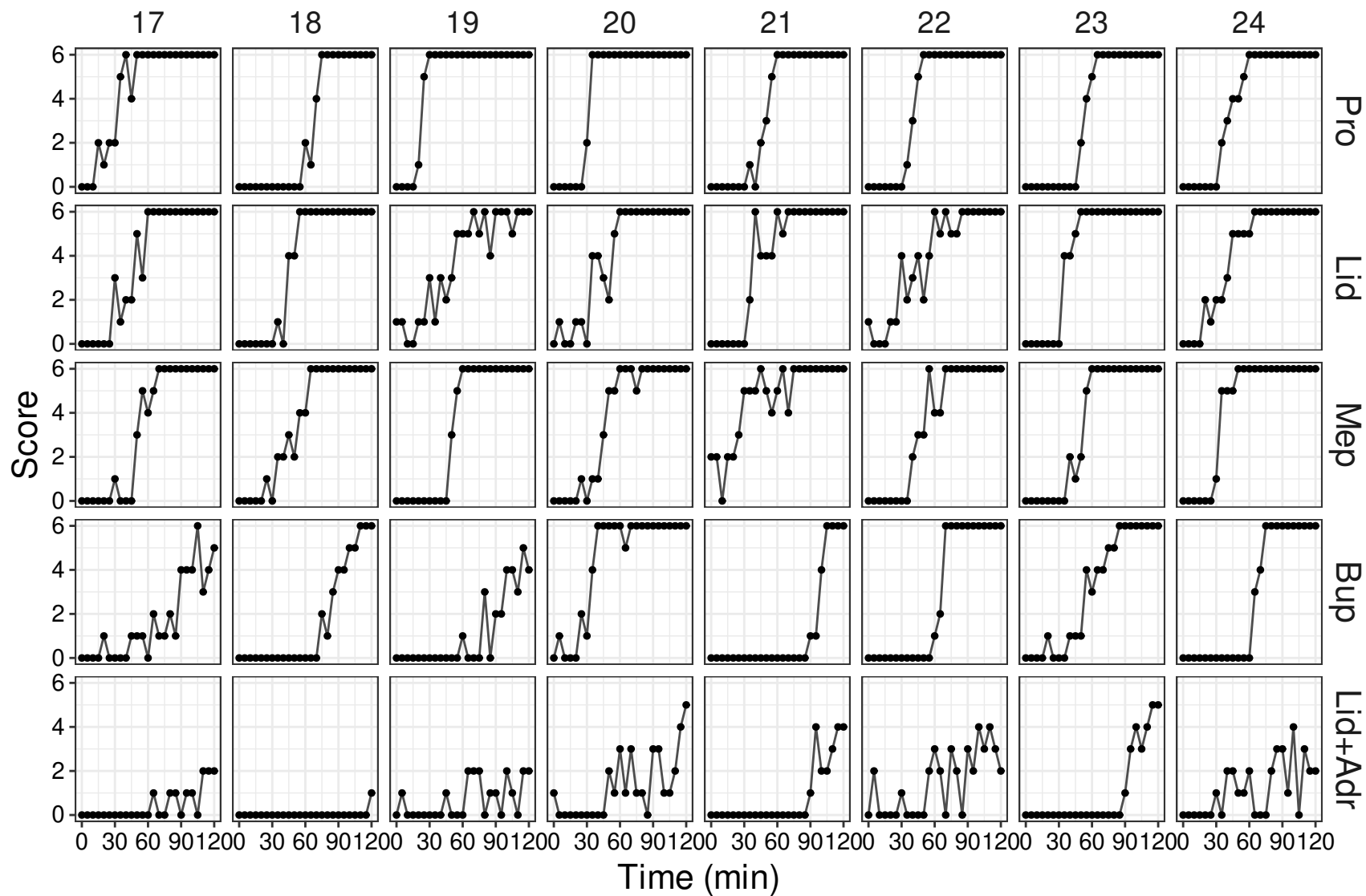

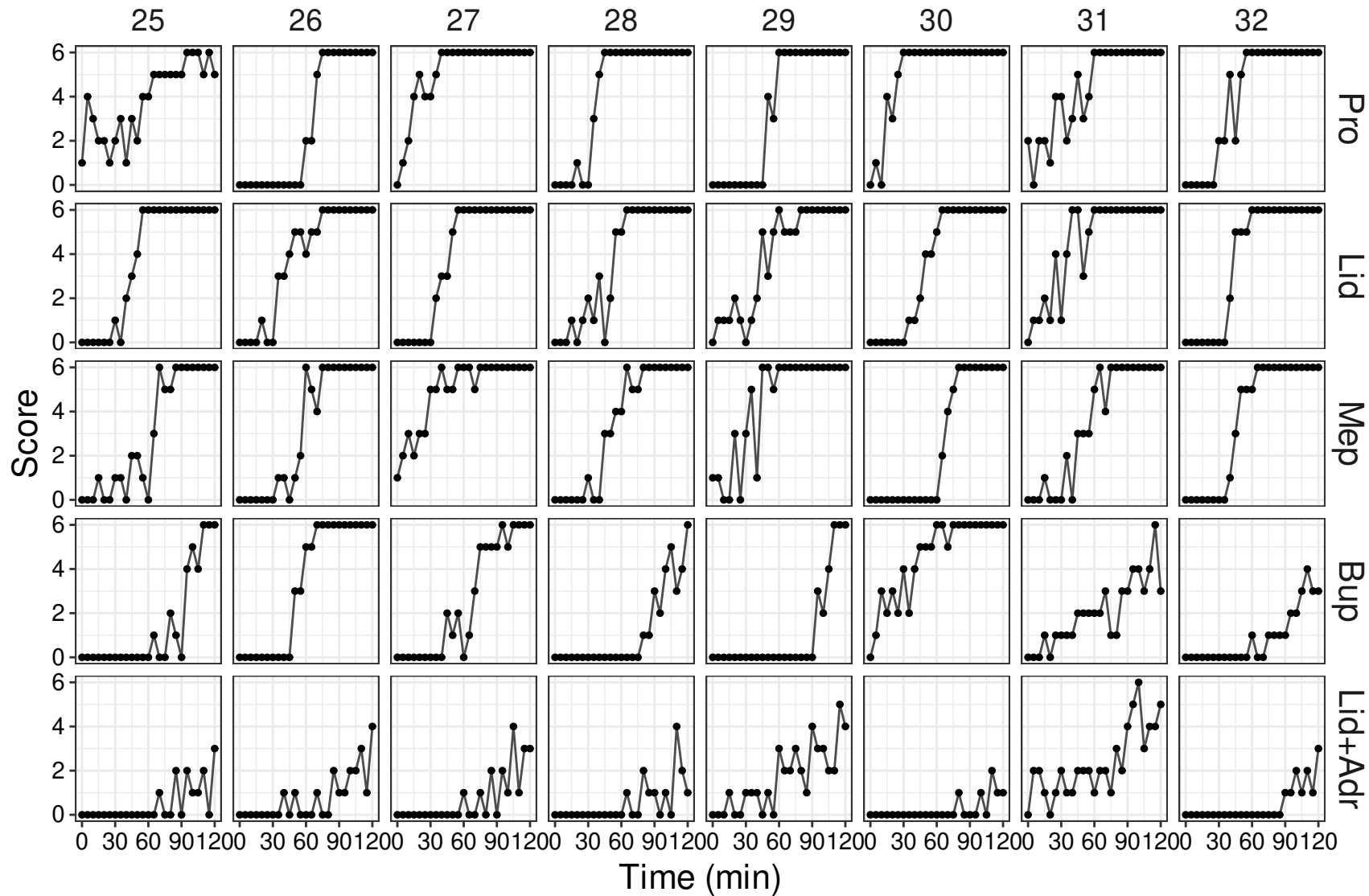

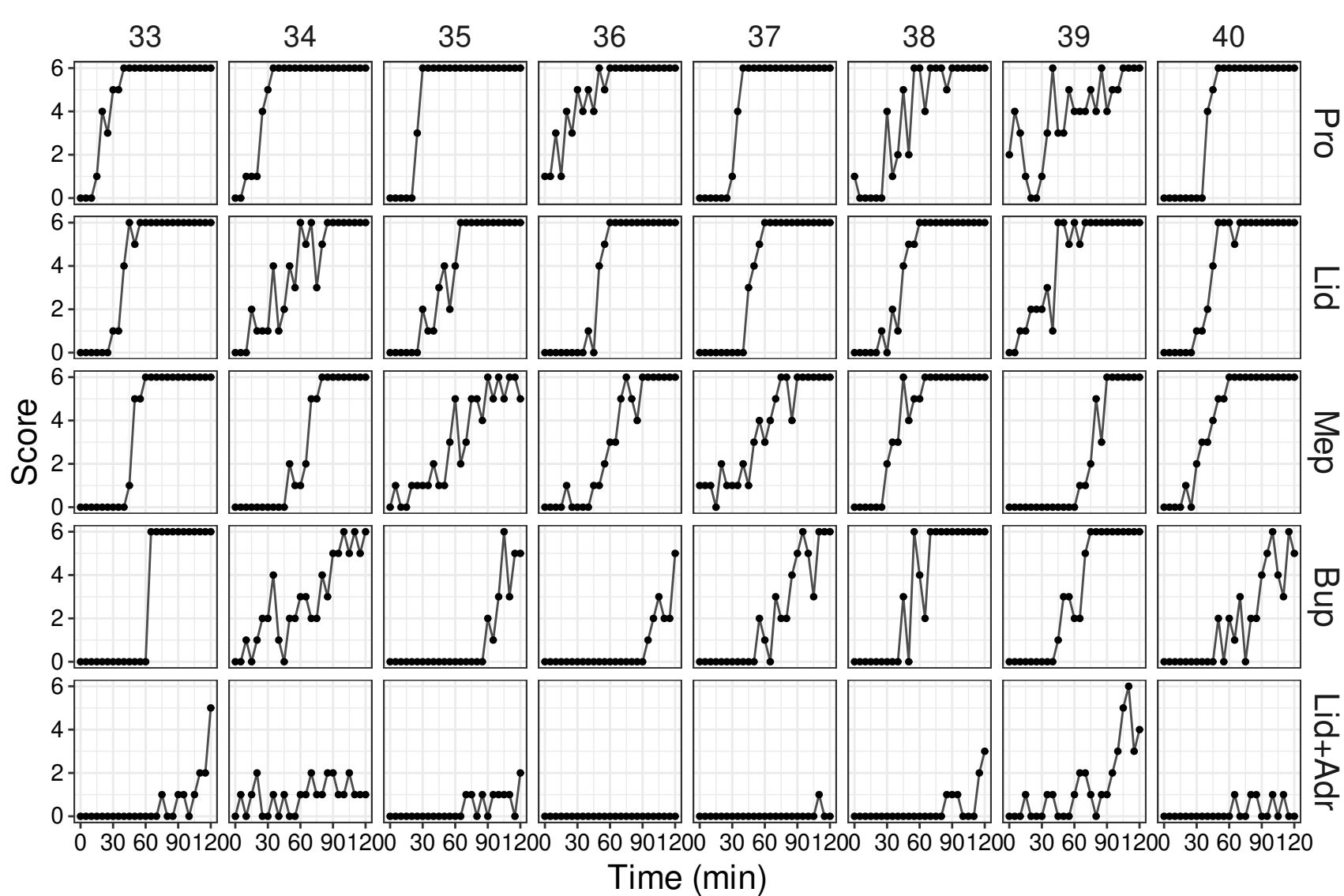

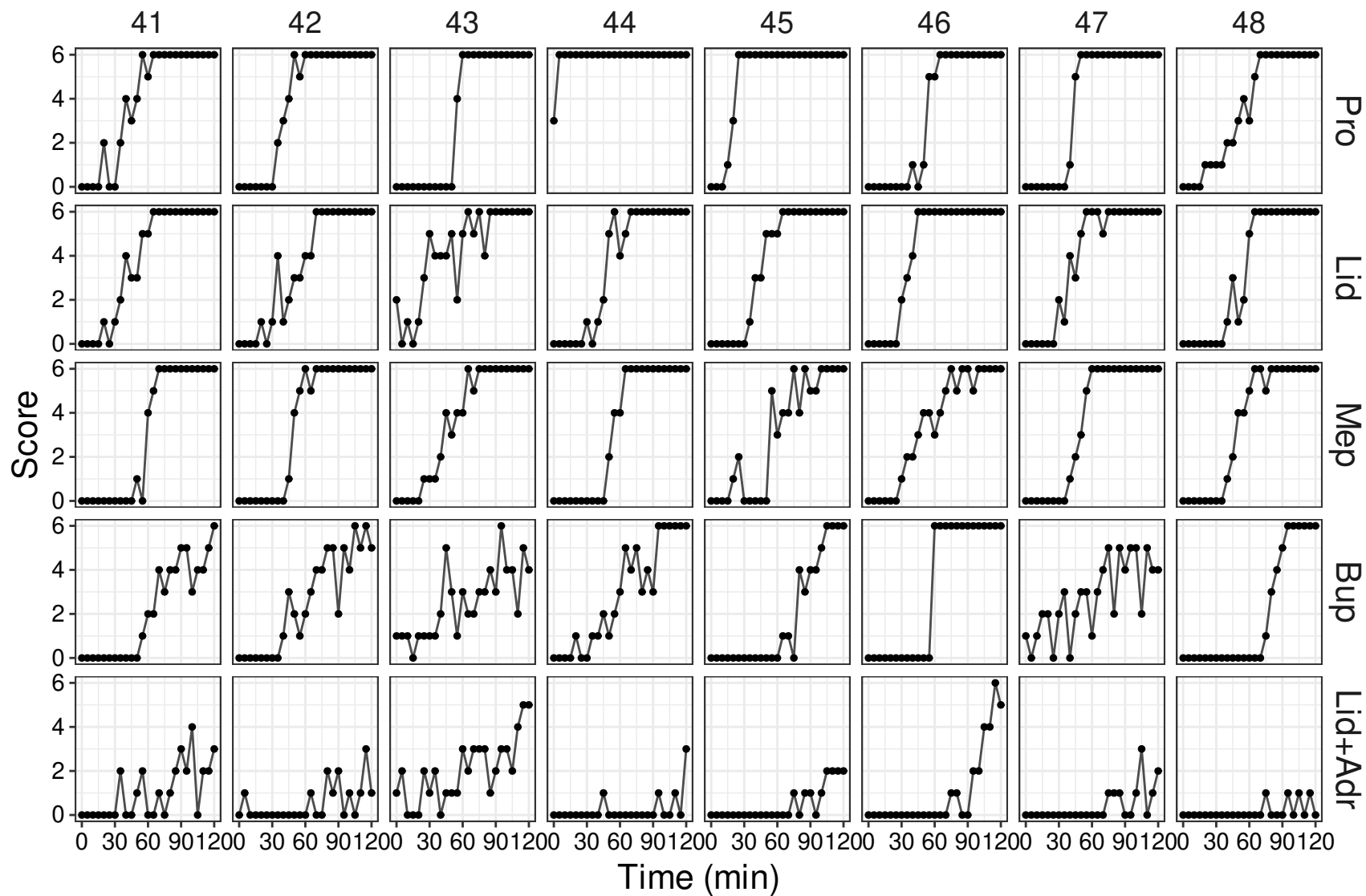

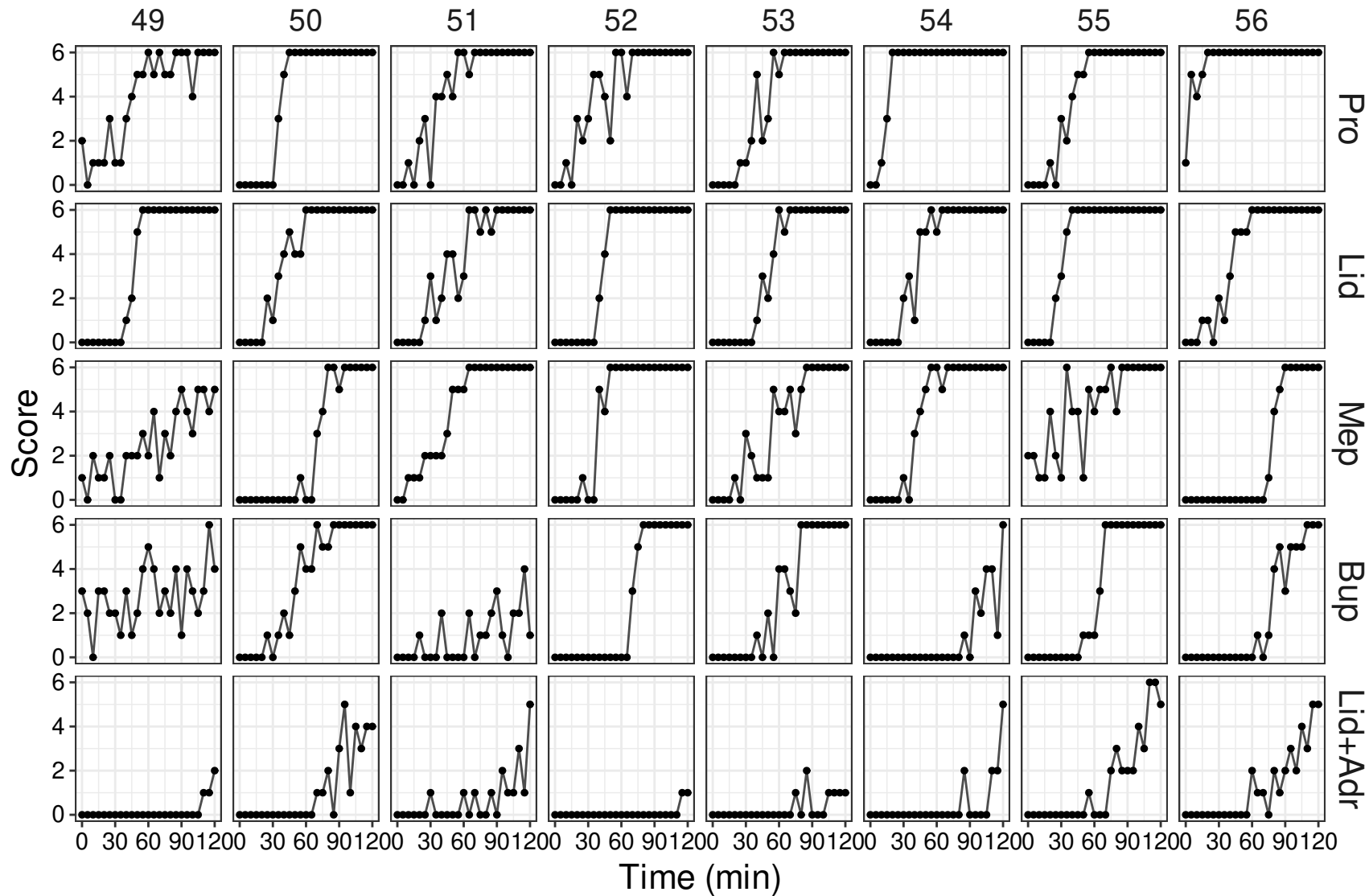

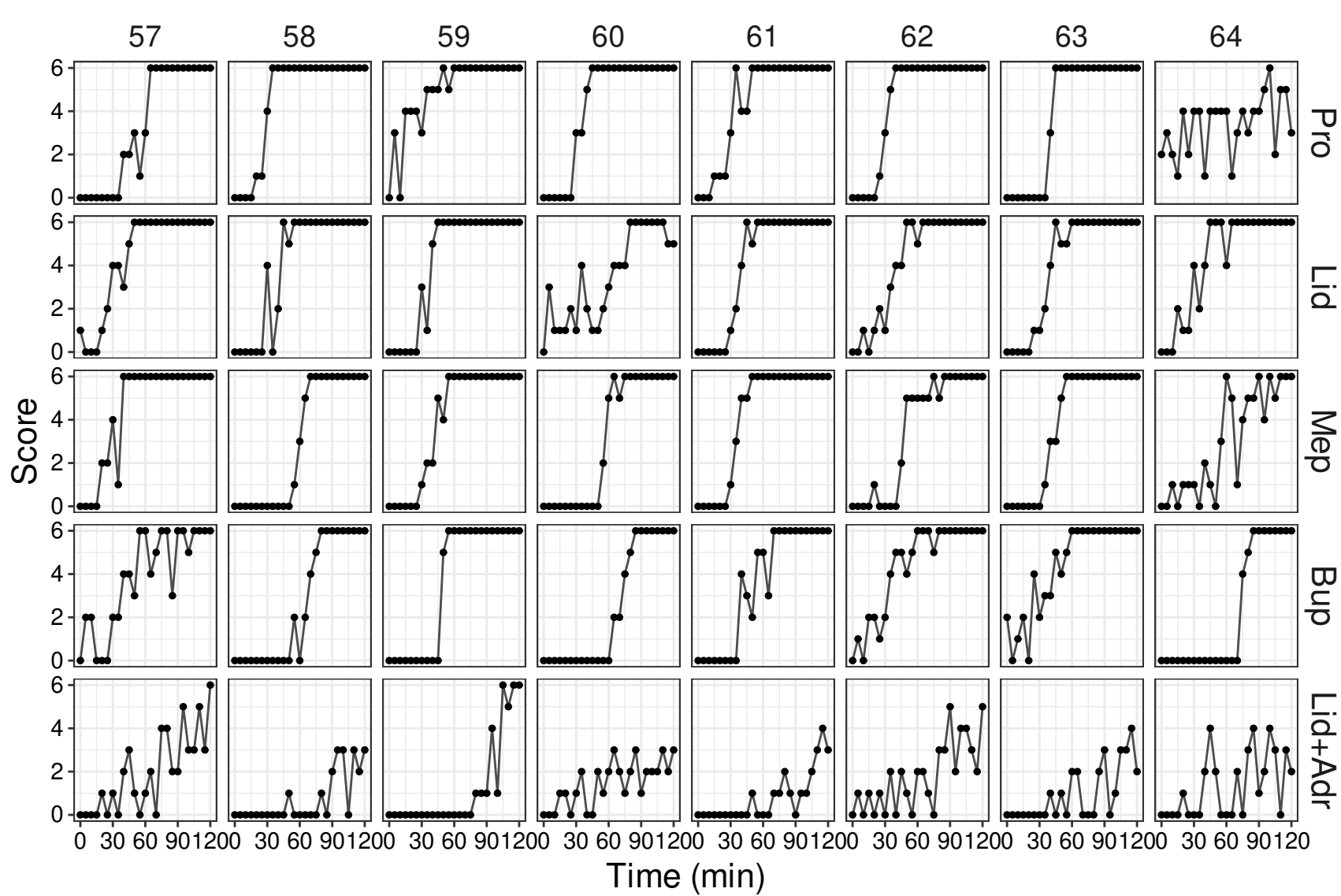

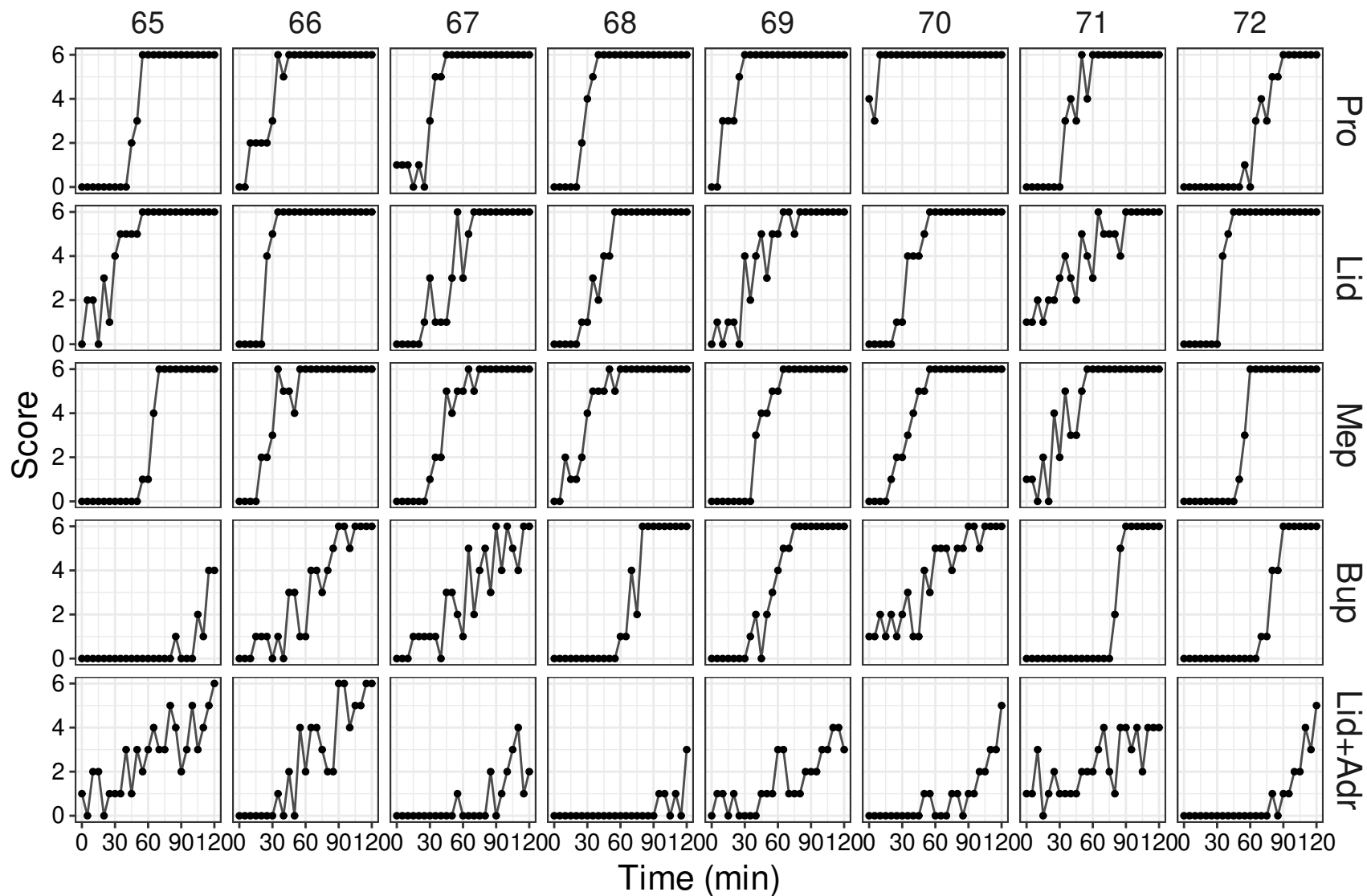

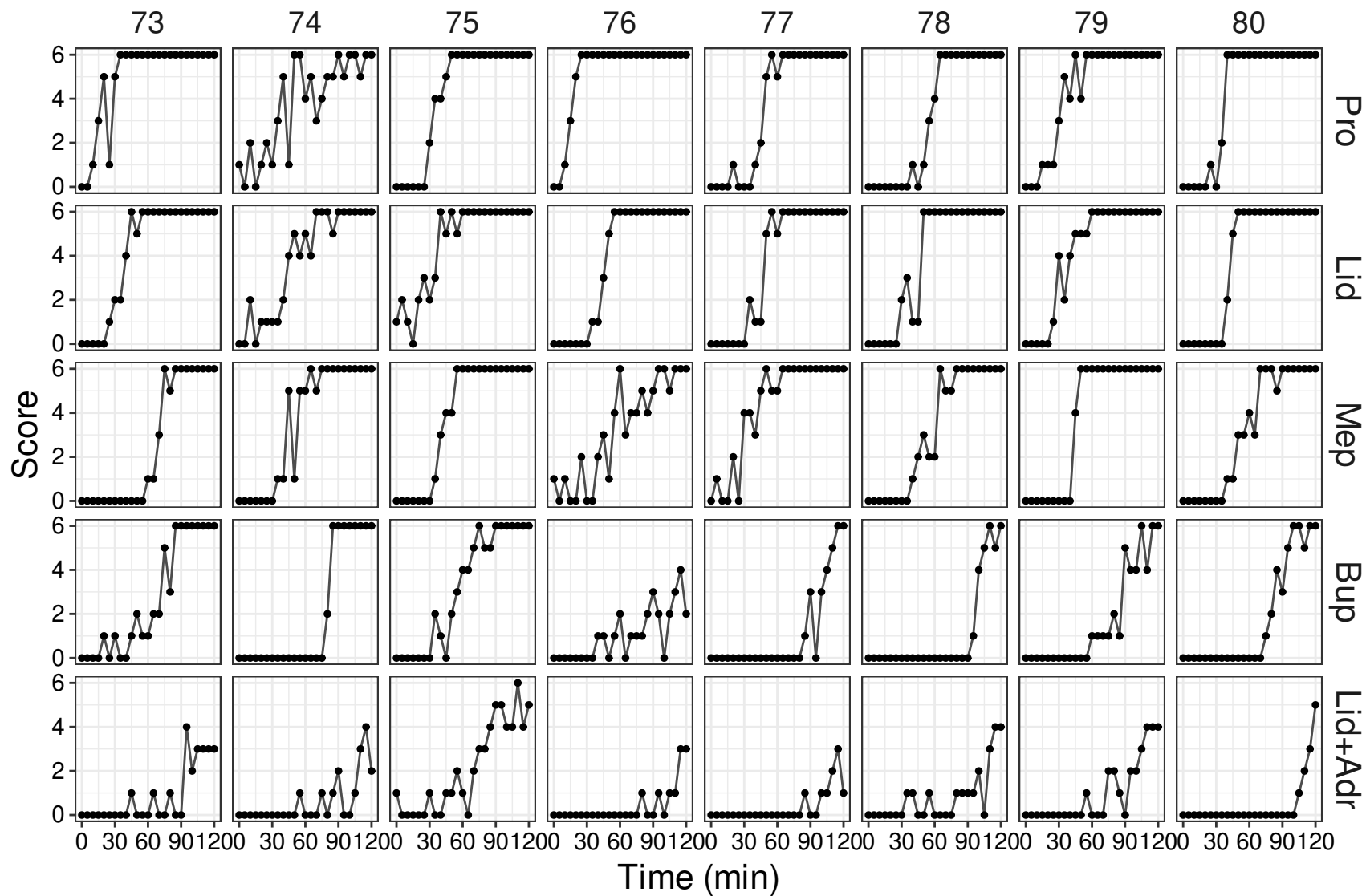

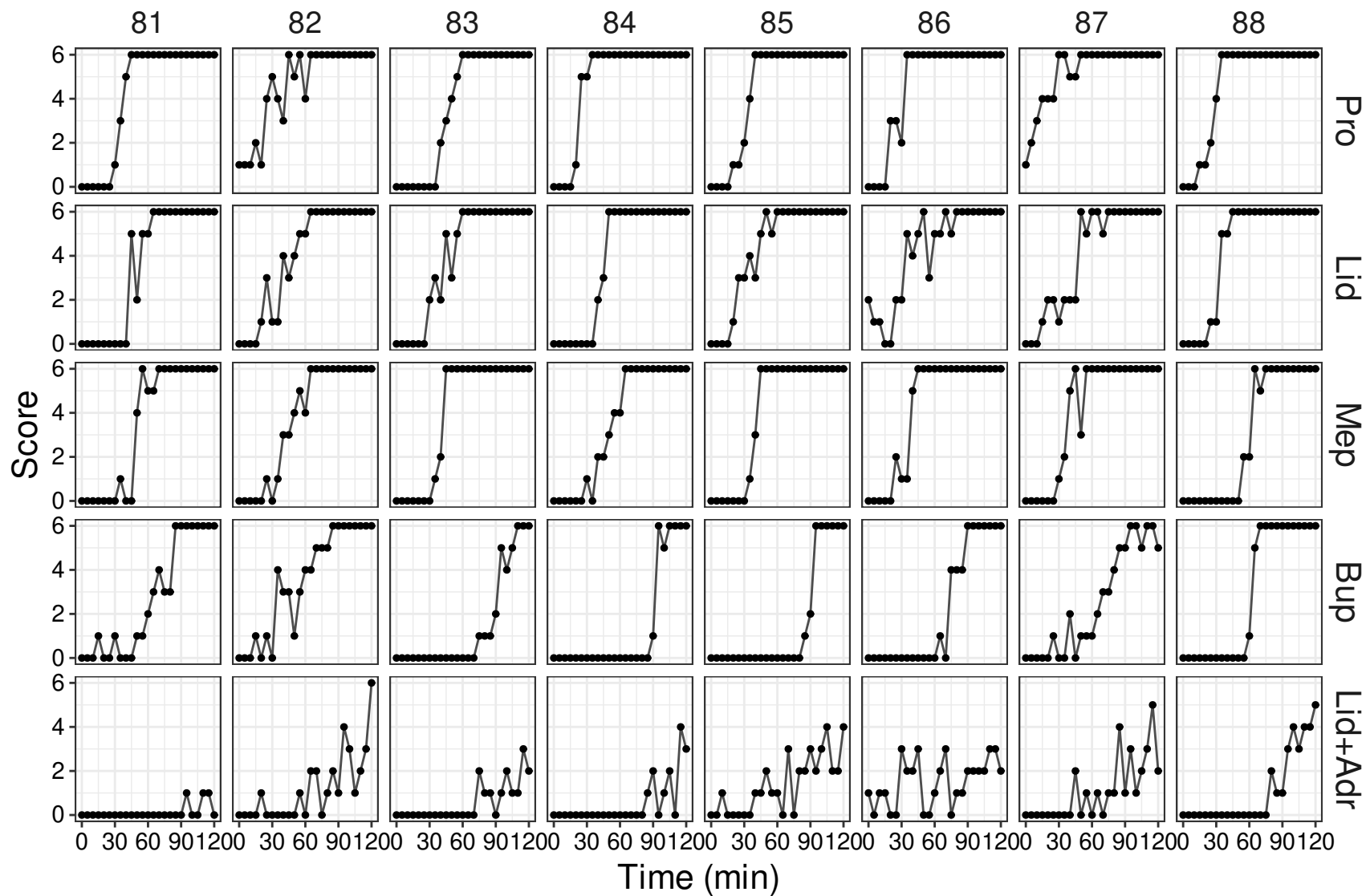

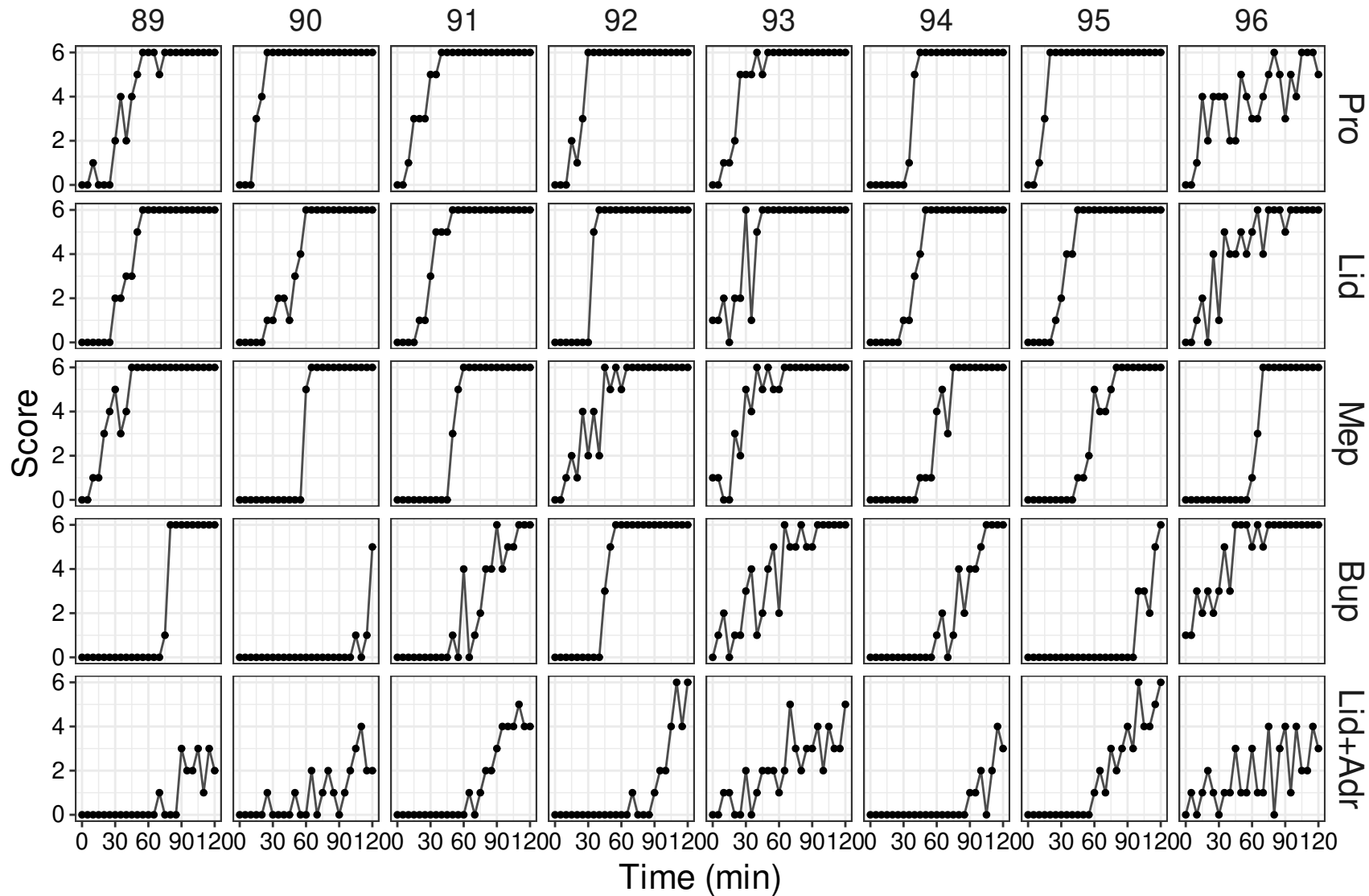

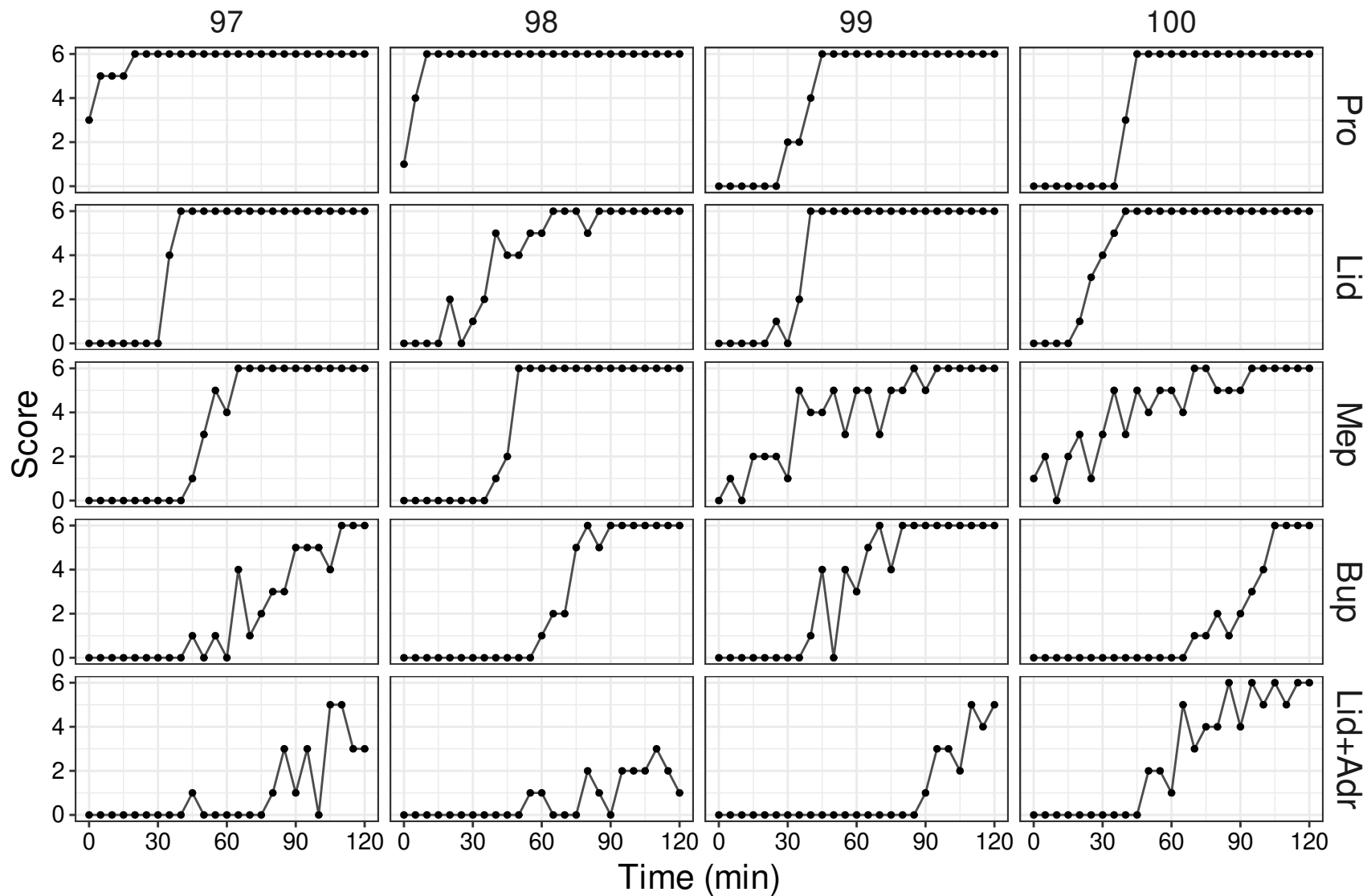

Supplement: Supplementary file 1 [file medicines-10-00061-s001.zip › SFig4.pdf]

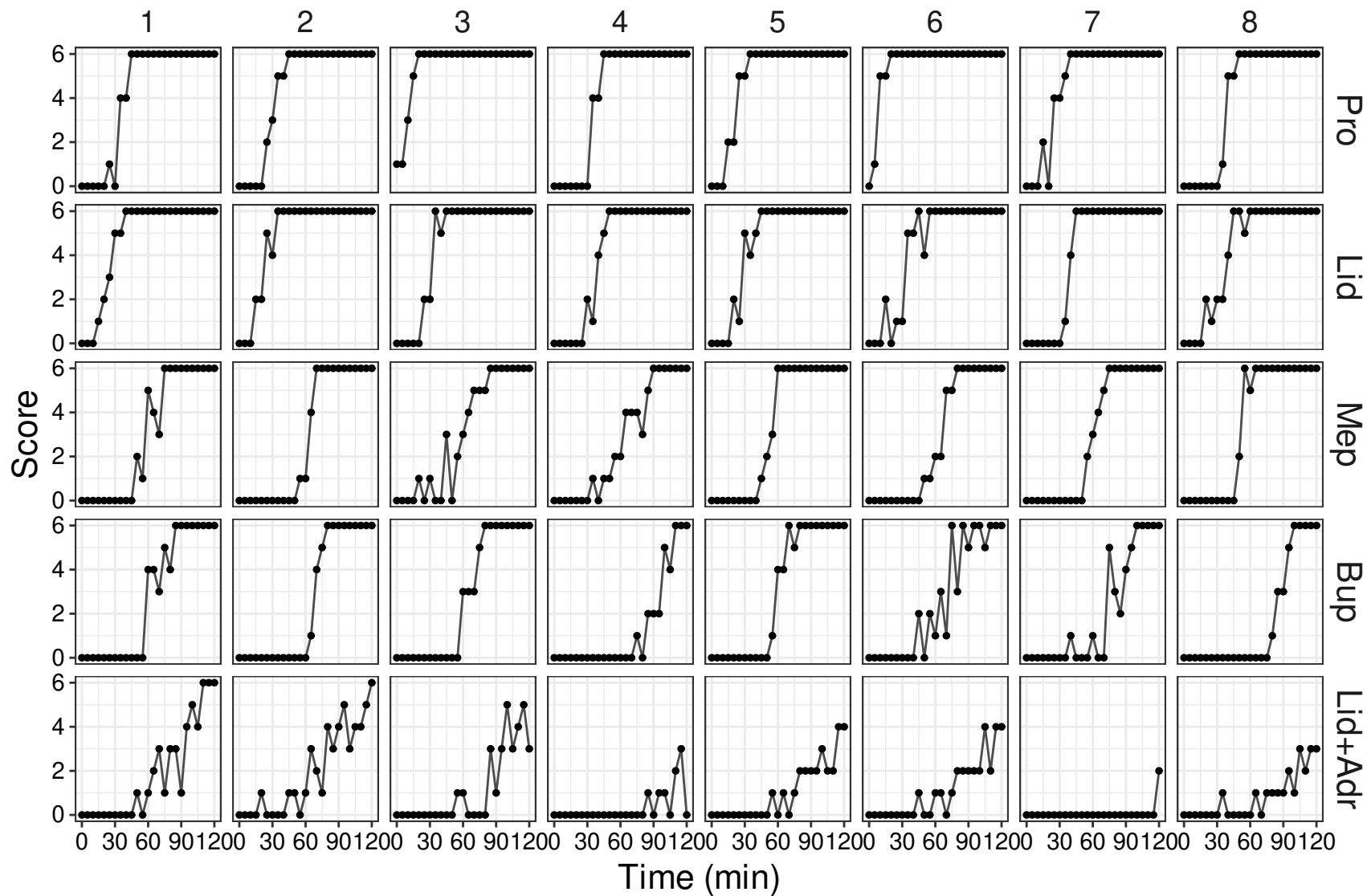

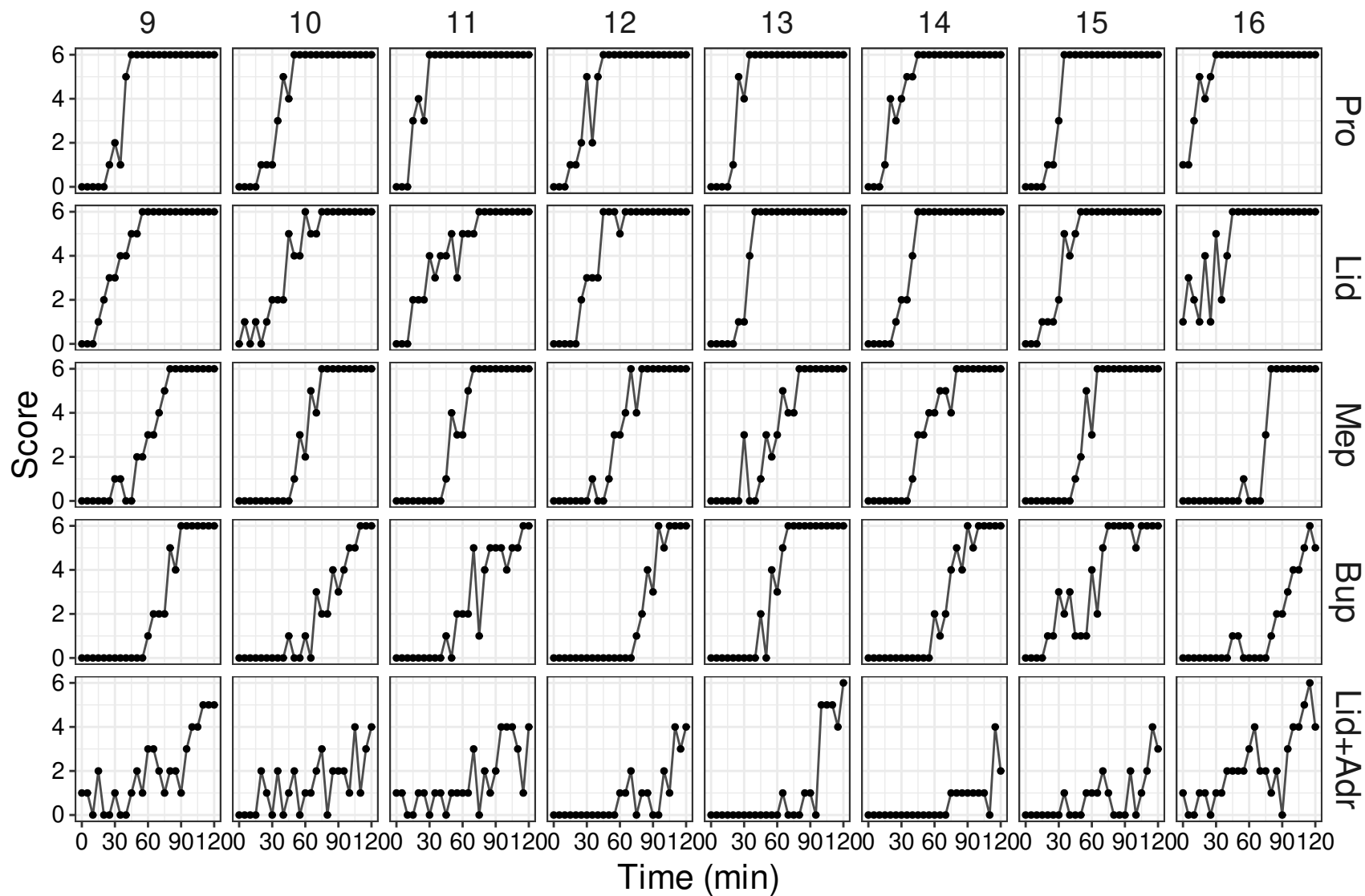

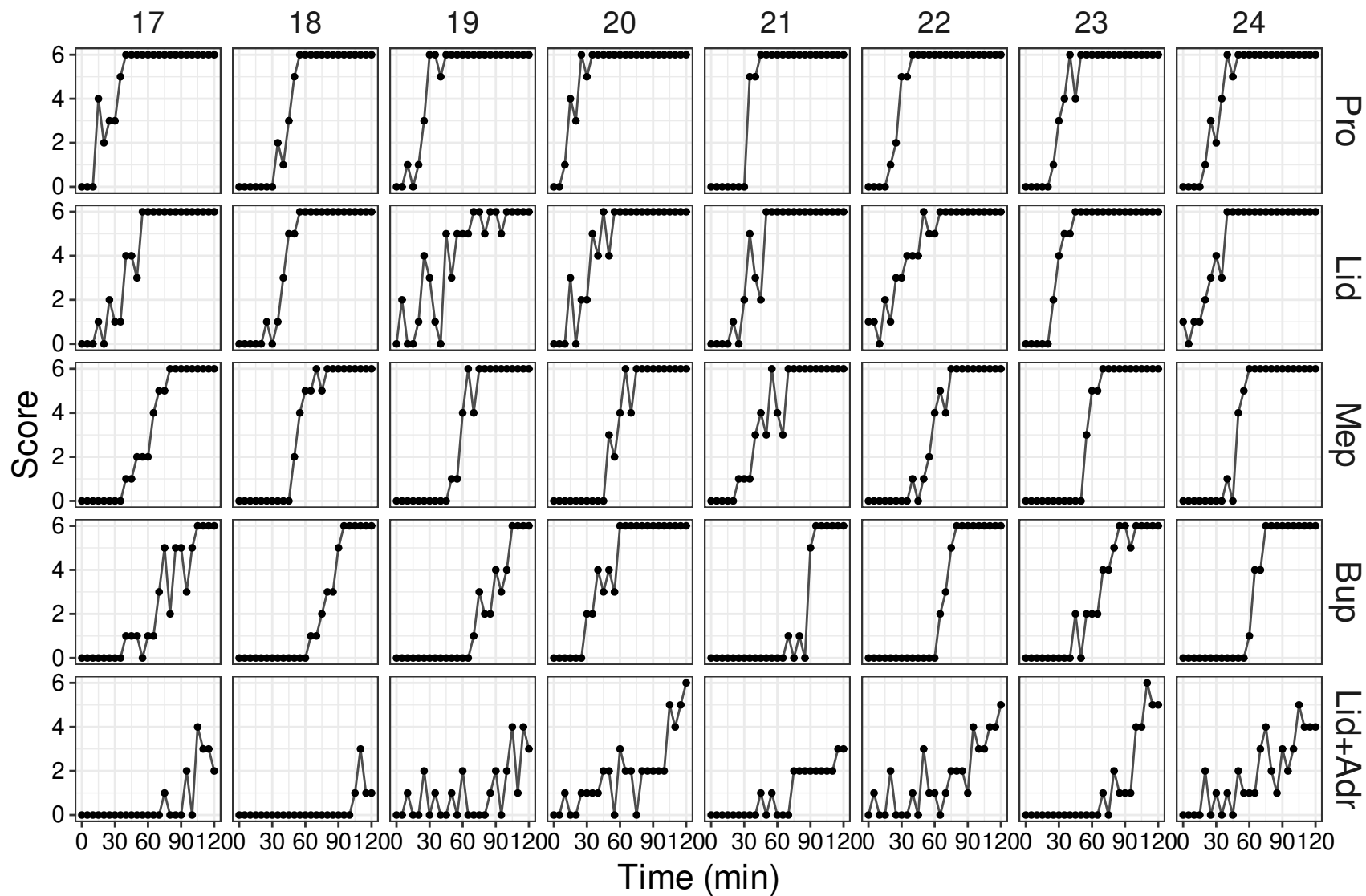

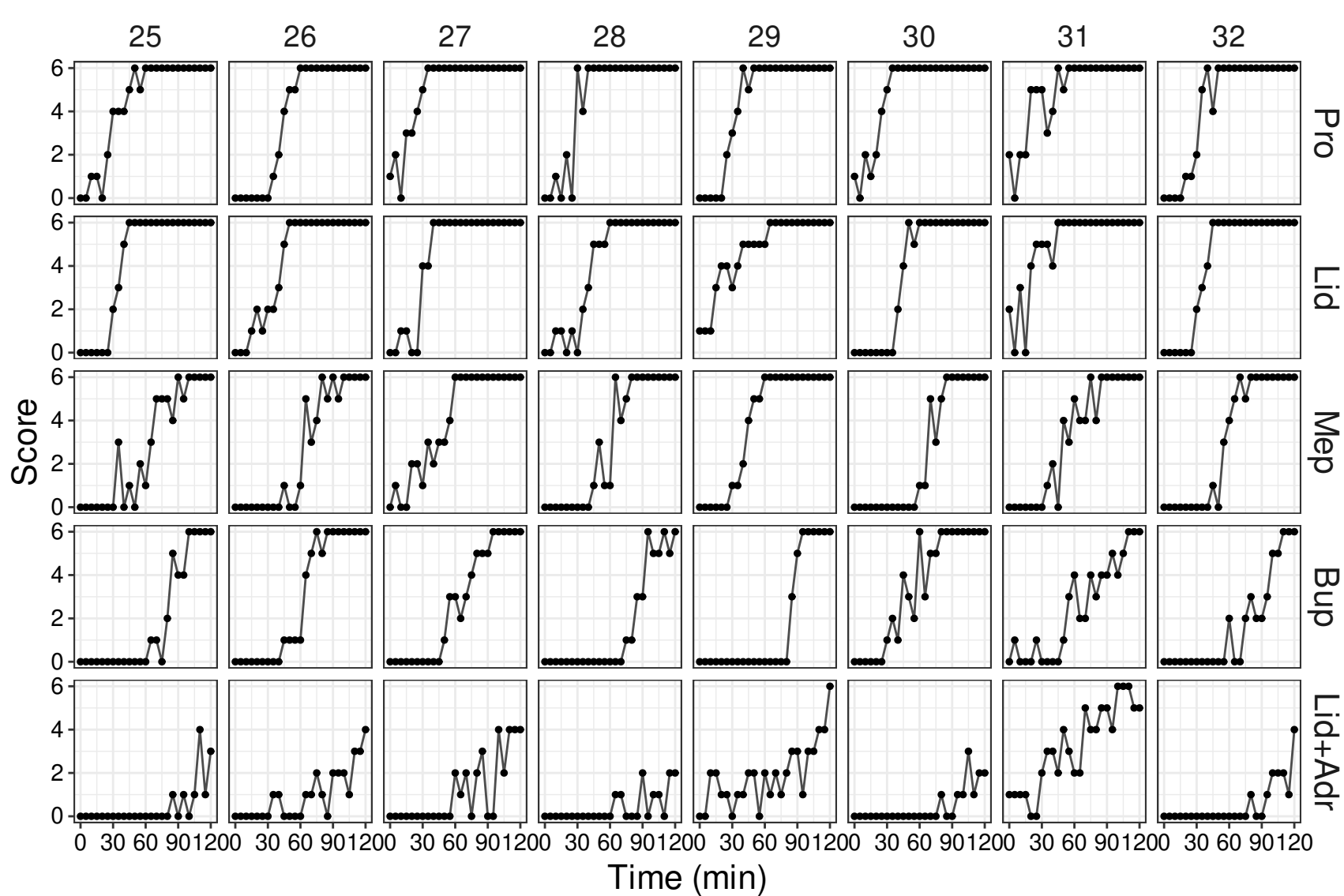

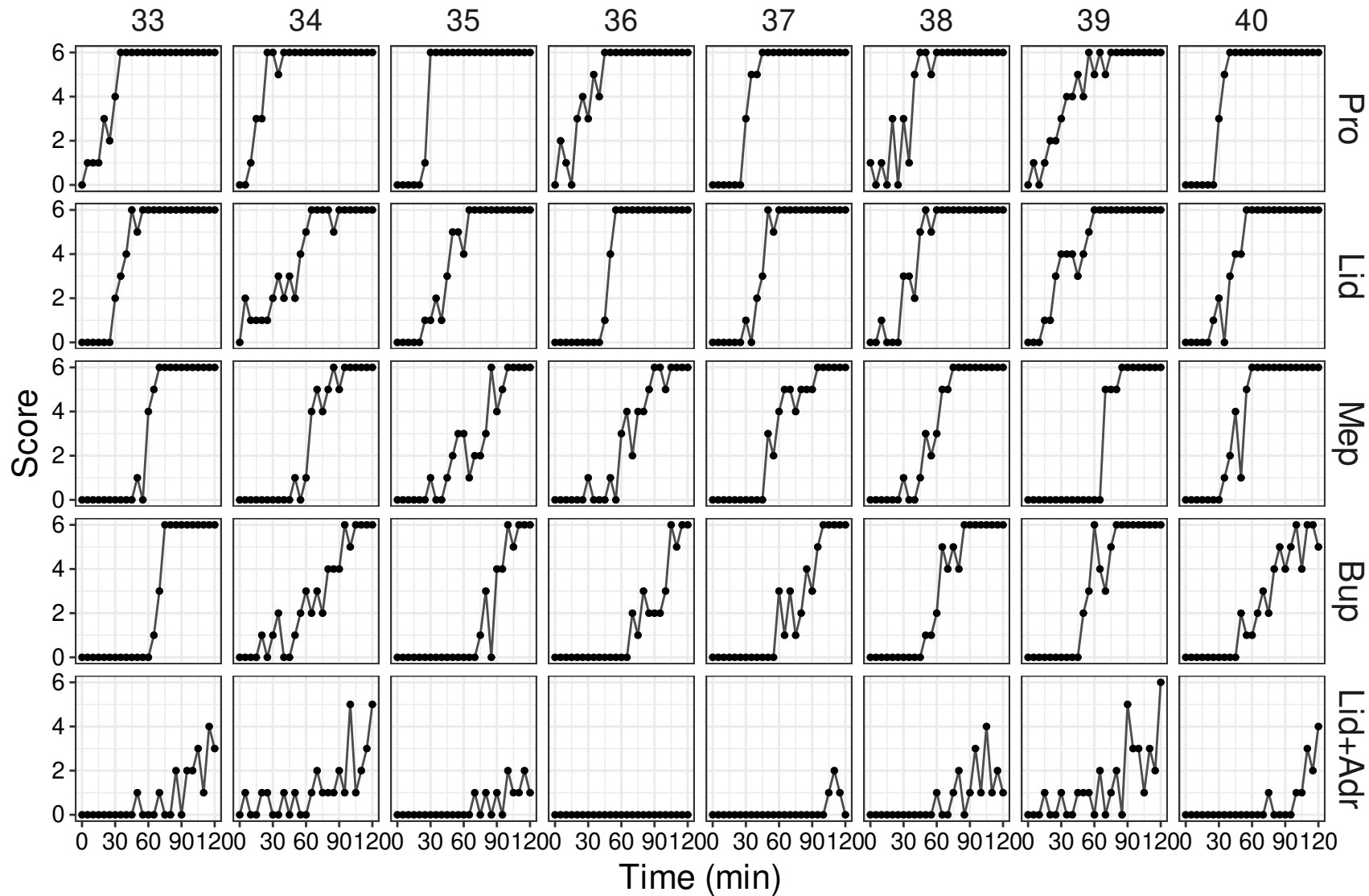

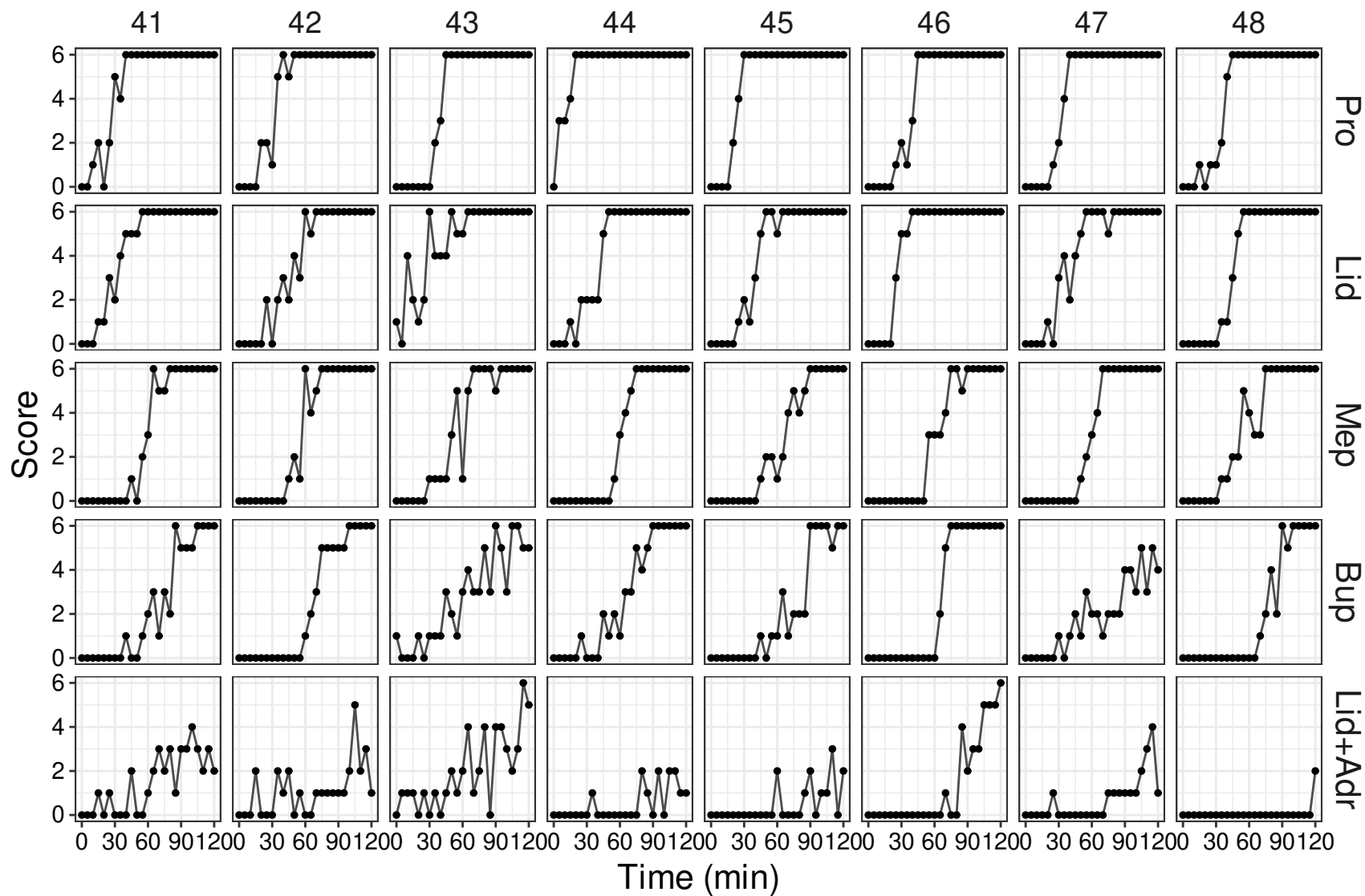

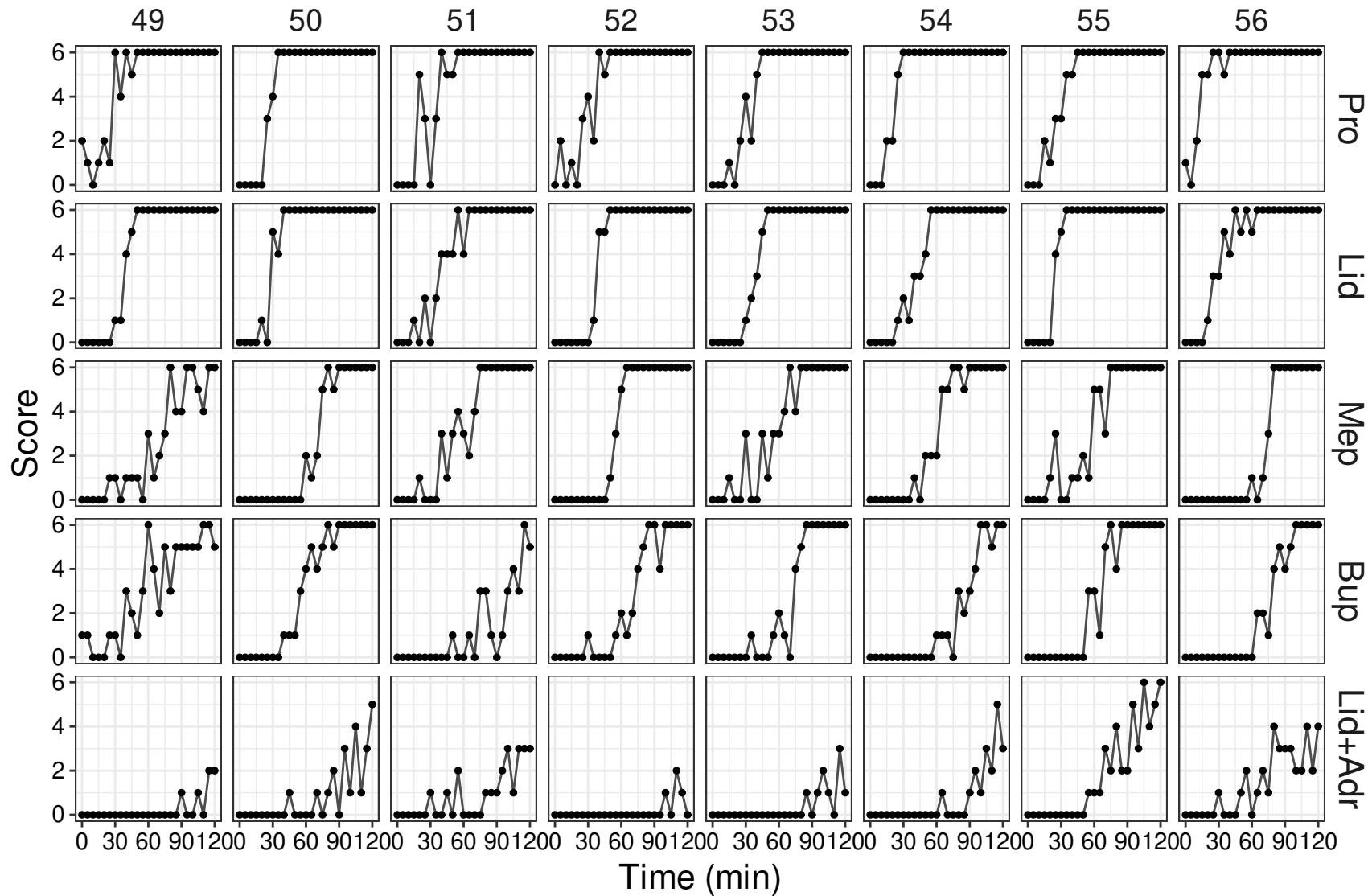

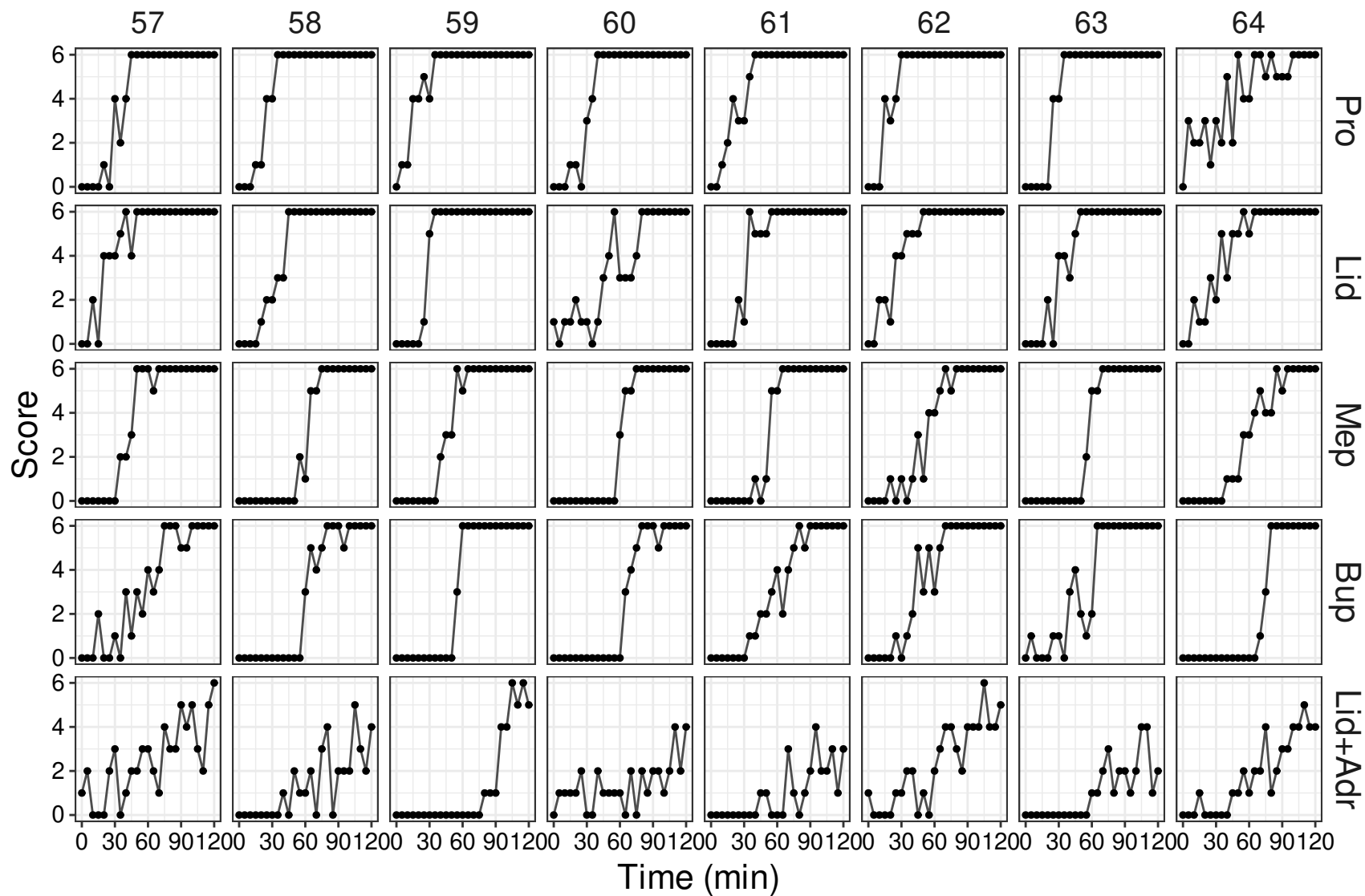

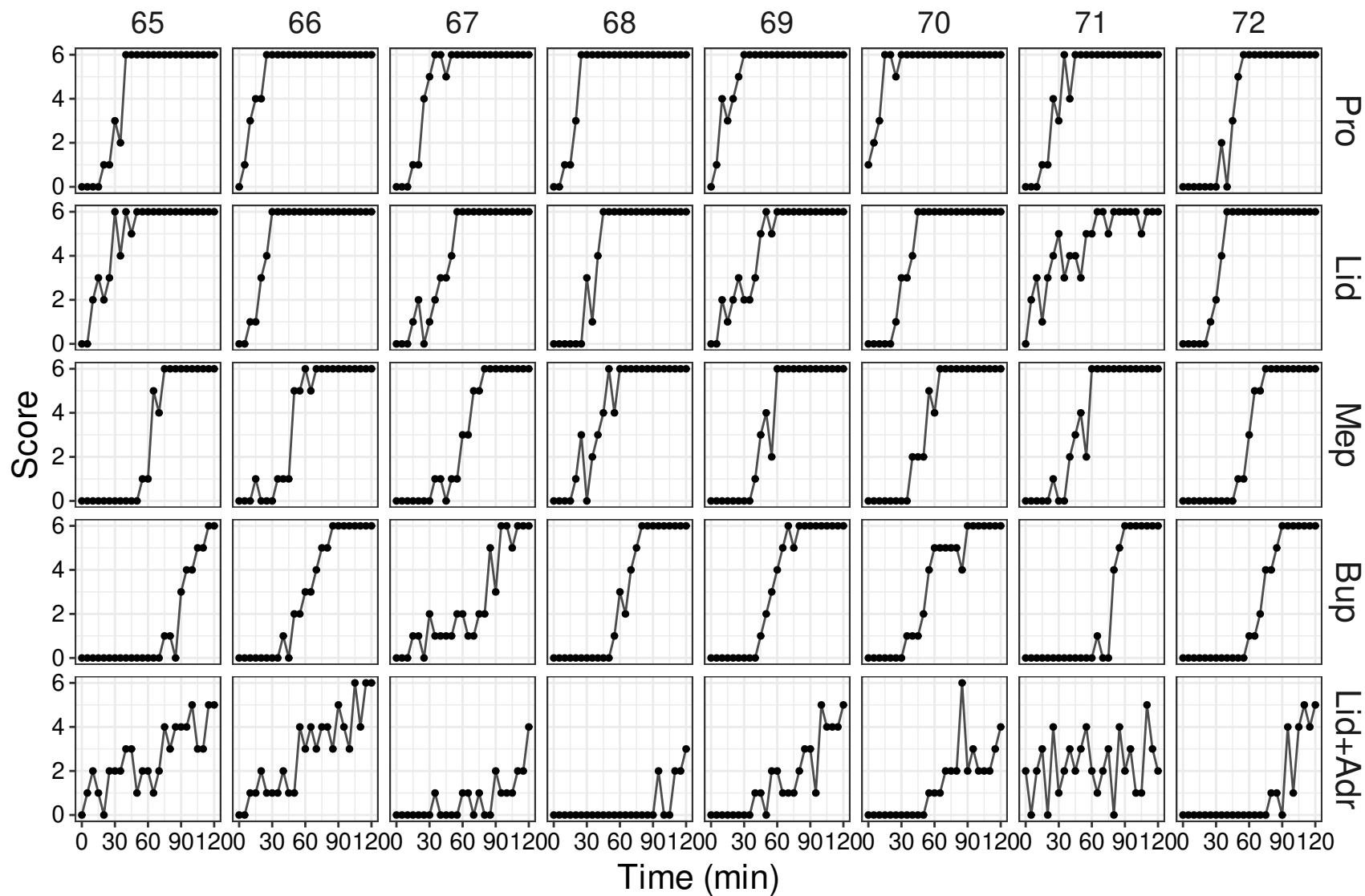

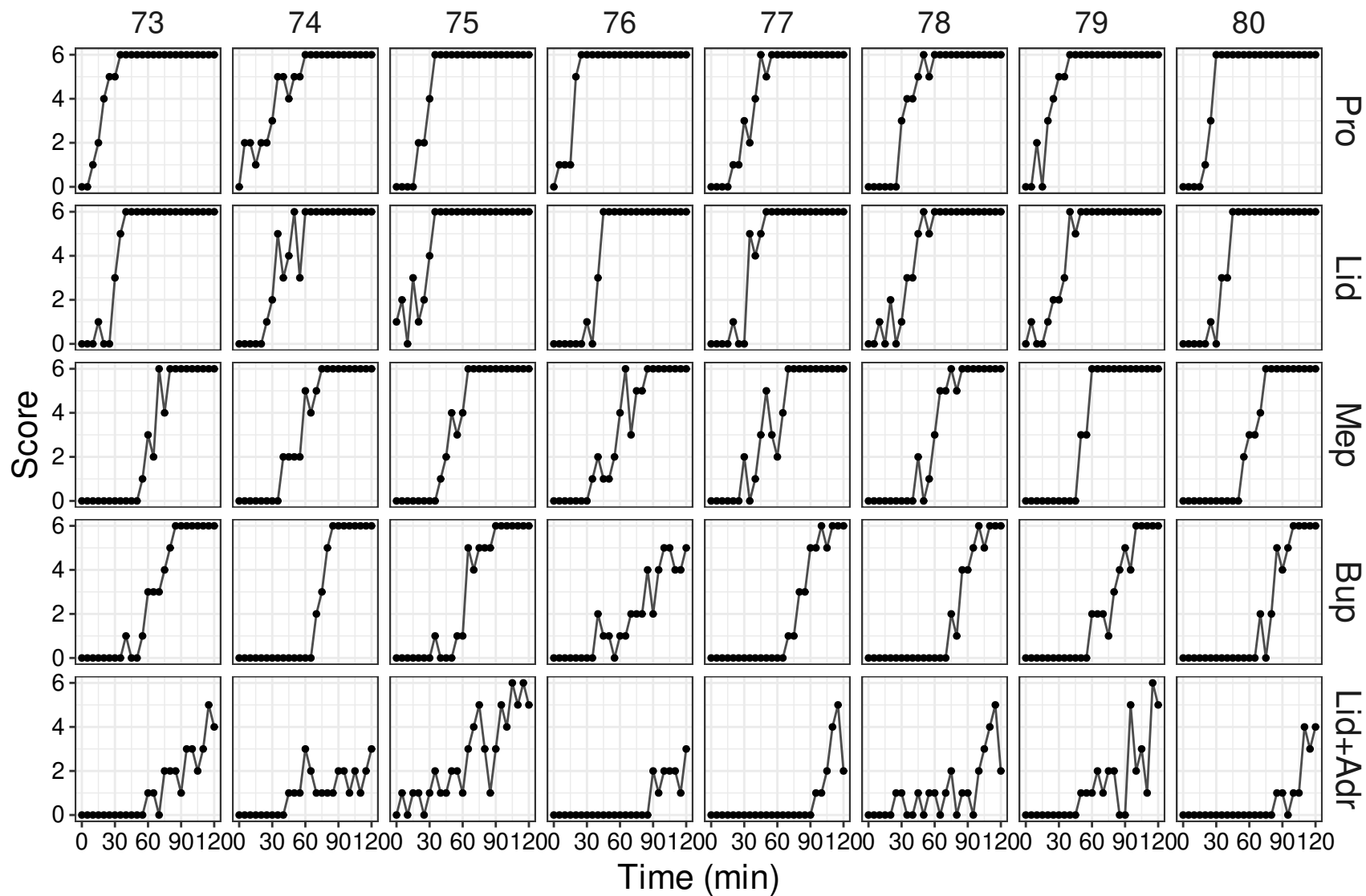

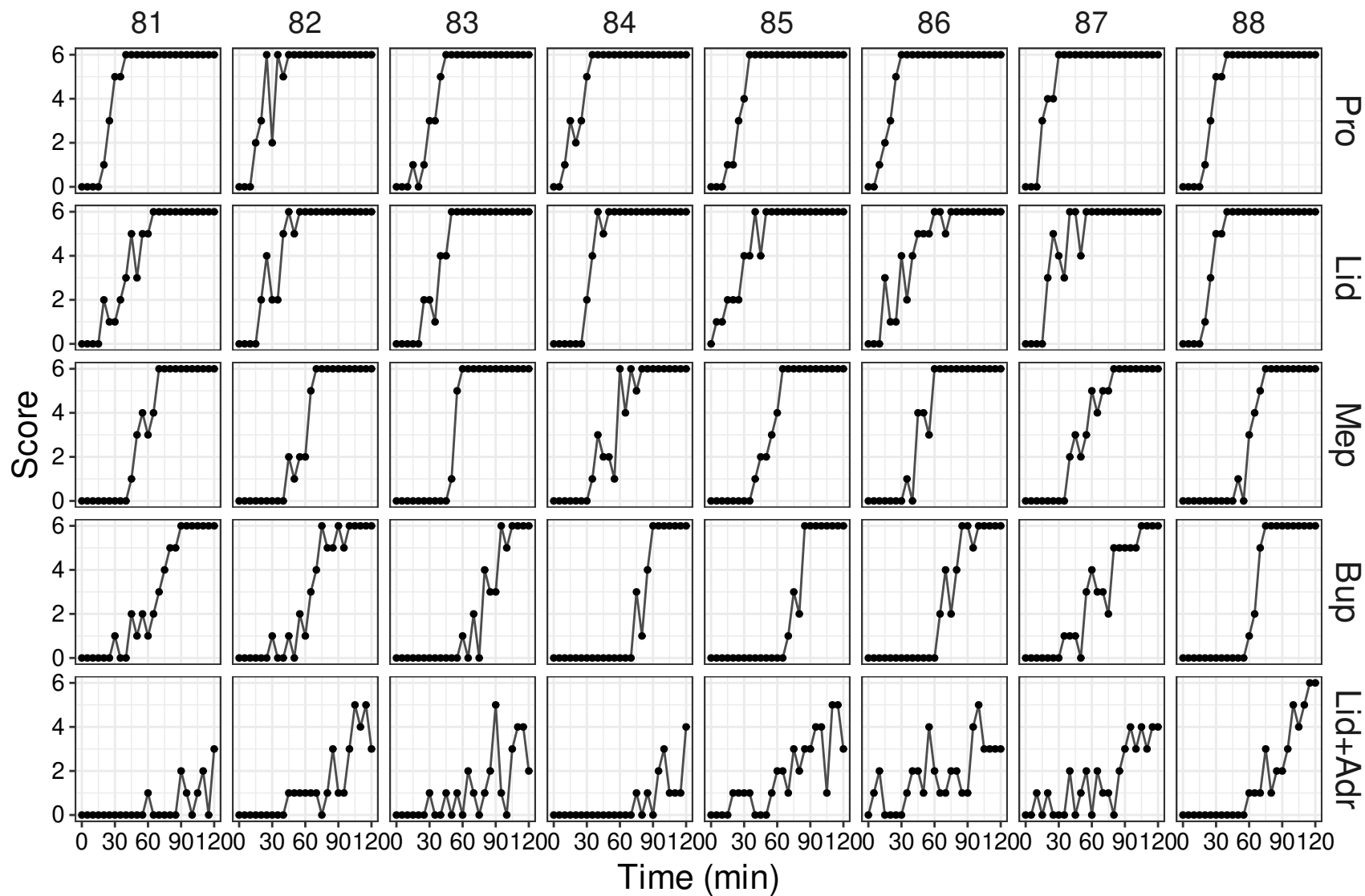

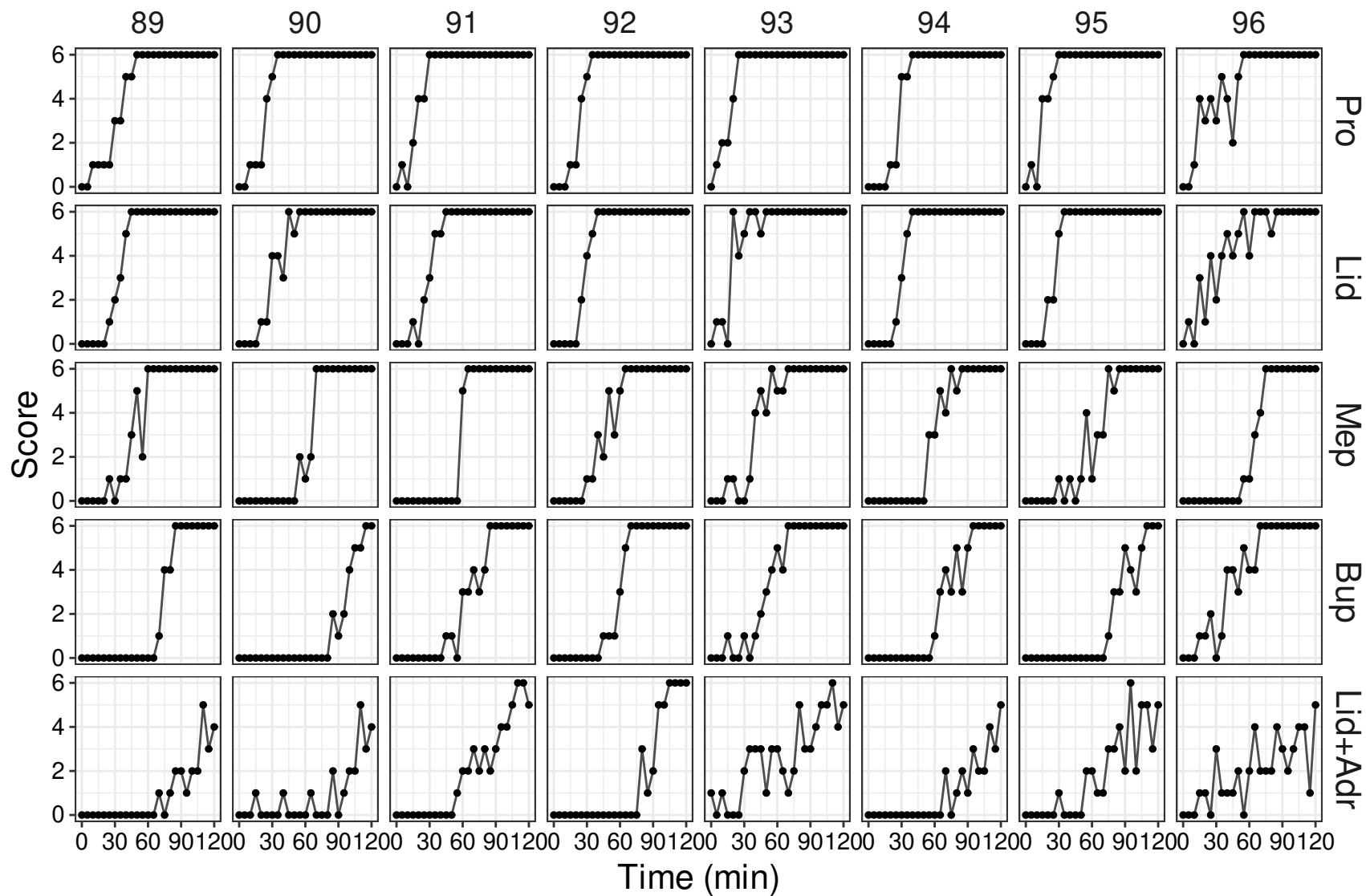

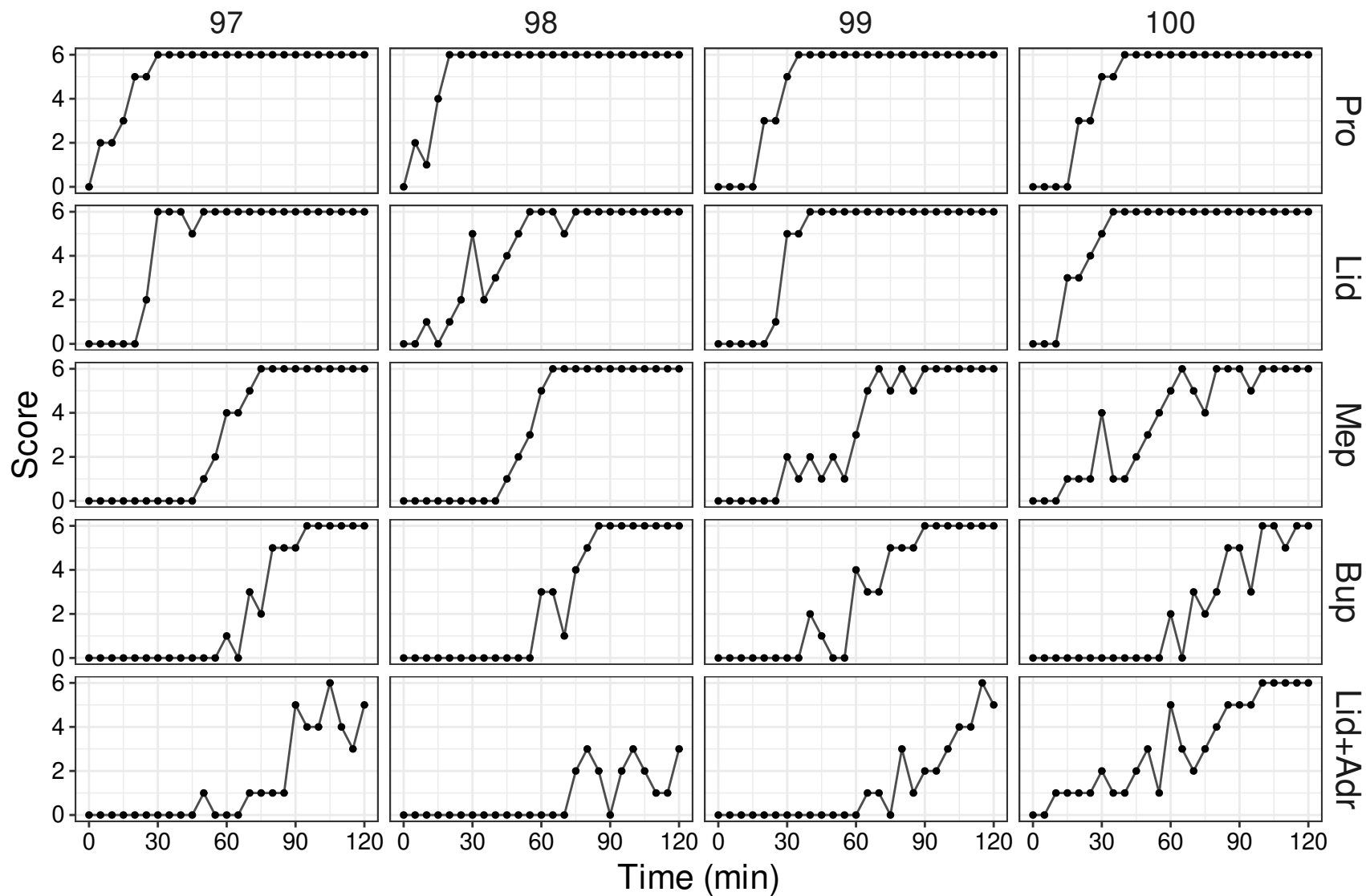

Supplement: Supplementary file 1 [file medicines-10-00061-s001.zip › SFig5.pdf]
